# Fertile high-K magmatism and hydrothermal alteration zone associated with porphyry copper mineralization in Samra area, SE Sinai, Egypt

**DOI:** 10.1038/s41598-025-28051-0

**Published:** 2025-12-20

**Authors:** El Sayed Saber, Mohamed Abd El-Wahed, Ahmed El Sheikh, Abdelbaset Abudeif, Mohamed Attia

**Affiliations:** 1 Geology Department, Faculty of Science, Sohag University, Sohag, 82524 Egypt; 2 Geology Department, Faculty of Science, Tanta University, P.O. Box: 31527, Tanta, Egypt; 3 State Key Laboratory of Continental Dynamics, Department of Geology, Northwest University, Xi’an, 710069 China; 4 Geology Department, Faculty of Science, Kafr El Sheikh University, Kafr El Sheikh, P.O. Box: 33511, Egypt

**Keywords:** Tarr complex, Wadi samra, Porphyry copper, Hydrothermal alteration, Southern sinai. fertile granitoids, Magnetite-series, I-type granites, Moderate oxidation magma, Environmental sciences, Solid Earth sciences

## Abstract

This study combines remote sensing and geochemical data to evaluate the porphyry copper mineralization in the Samra region of southeastern Sinai, Egypt. The Wadi Samra area, located within the Kid metamorphic belt and Tarr Complex, comprises volcanic flows, pyroclastics, breccias, tuffs, mudstones, schists, and albitic intrusions. These rocks are intruded by high-K calc-alkaline granitoids. The study utilizes Landsat-8 spectral bands and ASTER data to analyze the distribution of ferrous and ferric iron oxides within copper belts. The Tarr Complex, located in the Wadi Samra area, is characterized by three distinct deformation phases, with thrust faults dipping toward the northwest controlling the contacts among rhyodacitic tuffs, pyroclastics, albitite, and porphyritic dacite. Porphyry copper mineralization in the Samra area of southeastern Sinai occurs within a volcano-sedimentary sequence intruded by high-K calc-alkaline granitoids. Mineralization styles include quartz veins, stockworks, disseminated sulfides, and alteration zones associated with primary (pyrite, chalcopyrite, bornite) and secondary (malachite, azurite) copper minerals. The granitoids linked to this mineralization—mainly quartz-diorite and granodiorite—are classified as I-type, magnetite-series rocks formed from hydrous magma at temperatures between 800 and 900 °C and 20–30 km depths. These geochemical and petrological characteristics suggest favorable conditions for porphyry copper with minor gold mineralizations.

## Introduction

**Fig. 1:**
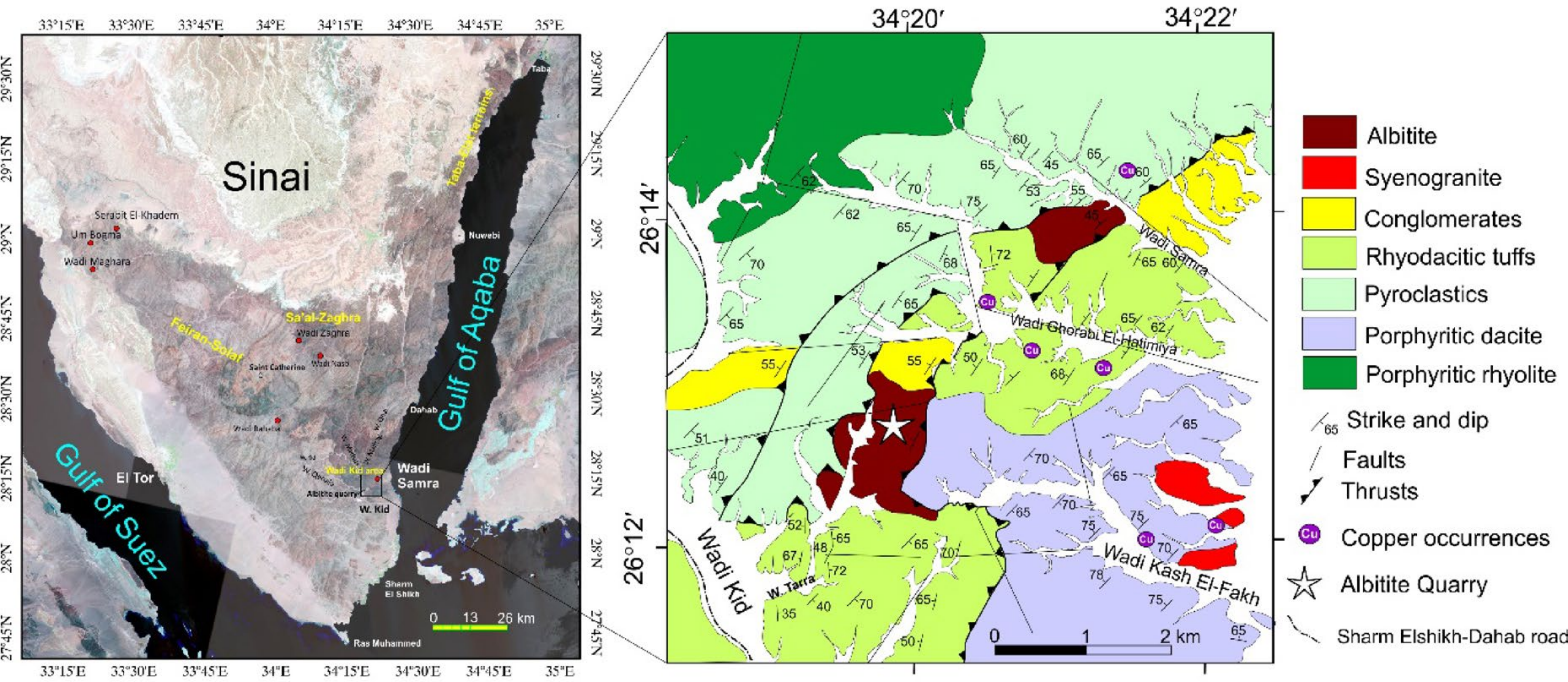
(**a**) Landsat image of Sinai showing locations of copper and the study area; (**b**) Geologic map of the study area (modified after Blasy et al.^32^ and Fowler et al.^43^; Fahmy et al.^54^). These figures were created and processed by ENVI v. 5.6.2. software: https://www.l3harrisgeospatial.com/Software-Technology/ENVI), which is mainly utilized for image processing, and ArcGIS Desktop 10.8. (https://www.esri.com/en-us/arcgis/products/arcgis-desktop/overview/).

The southern Sinai Peninsula comprises four major Neoproterozoic metamorphic blocks, among which the Wadi Kid terrane is a fundamental element (Fig. 1a). Together with the Feiran-Solaf, Sa’al-Zaghra, and Taba-Elat terranes, it represents the northernmost exposure of the East African Orogen in Egypt, forming the Egyptian Nubain Shield^1–4^,. The southwestern part of Sinai, Egypt, has historically been a key area for copper extraction, dating back to antiquity. Notable locations encompass Serabit El-Khadem, Wadi Nasb, Um Bogma, and Wadi Maghara (Fig. 1a). These sites are rich in copper mineralization found within Cambro-Ordovician and Carboniferous geological formations, frequently linked with manganese. Historical mining activities targeted copper and turquoise, indicating ancient smelting processes and workshops.

Copper mineralization in South Sinai is predominantly hosted by Precambrian igneous and metamorphic rocks, with mineralization also present in the overlying Paleozoic units, such as the Araba Formation (Cambro-Ordovician) and middle Cambrian layers. Copper mineralization in southern Sinai is structurally controlled, with significant mineralizations located in shear zones at Wadi Bat’hat Um Rabei, Wadi Rahaba, Wadi Samra, and Wadi El Regeita (Fig. 1a). These mineralizations are hosted within the Neoproterozoic crystalline basement. On the other hand, sedimentary-hosted copper-uranium mineralization in southwestern Sinai is confined to the Um Bogma Formation, with key occurrences at Um Bogma area, Sarabeet El Khadim, and Wadi Nasib. These appear as carbonate-heavy weathered soils, rich in copper and uranium minerals, demonstrating effective copper extraction rates through leaching^5–8^. Copper occurrences in sedimentary formations originate from both telodiagenetic and epigenetic-supergene processes. Those of telodiagenetic origin were formed during early and mesodiagenetic stages under reducing conditions. Metal sources for these mineralizations include remobilized components from clastic units, the crystalline basement, and proximal porphyry systems. Epigenetic-supergene mineralizations formed through the movement of metal-rich hydrothermal fluids—enriched in iron, manganese, sulfur, and copper—along fault systems and within karst-altered carbonate units of the Um Bogma Formation^5^.

Copper mineralization associated with igneous and metamorphic rocks includes that at Wadi El Regeita, Kid ring structure, Wadi Rofaiyed, Wadi Samra, and Gabal Tarr. Copper mineralization at Wadi El Regeita occurs primarily within monzogranite host rocks, with localized enrichment in hydrothermally altered andesitic dykes that served as conduits for mineralizing fluids^9^. At the Kid Ring Structure, the presence of Cu-Au-Zn associations linked to volcanic activities and granitic intrusions^10^. At Wadi Rofaiyed Area, the copper mineralization is classified as porphyry-type, formed during a large-scale extensional tectonic event following the collisional period^11^. Copper mineralization in all these areas has been exploited and subjected to mining activities since the predynastic times^6–8^. Historical records indicate that mining operations in ancient South Sinai primarily targeted copper extraction, with archaeological discoveries revealing mining locations and smelting facilities. These locations have been associated with substantial copper production in antiquity, especially in regions such as Wadi Nasb, which functioned as a hub for copper processing.

The application of multispectral satellite data in mineral exploration is especially useful in remote and difficult-to-access metallogenic regions, aiding mining firms and exploration geologists during early-stage reconnaissance surveys.

The visible to thermal infrared regions of multispectral sensors—specifically VNIR, SWIR, and TIR bands from platforms such as Landsat-8, ASTER, and Sentinel-2—offer robust capabilities for identifying hydrothermal alteration assemblages and distinguishing lithological units linked to mineralized systems, while also supporting structural and stratigraphic mapping^12–23^.

Moderate-resolution multispectral imagery has demonstrated effectiveness in regional-scale exploration for Au, Cu, Zn, and Pb, particularly across the Arabian–Nubian Shield, including the Sinai Peninsula and Egypt’s Eastern Desert^12–17,21,24^ .

Within the Kid Metamorphic Belt of late Precambrian age, the Wadi Samra region is identified as a major metallogenic domain with significant copper and gold mineralization.

Secondary copper minerals, such as malachite and chrysocolla, occur as supergene alteration products formed by the oxidative weathering of primary sulfides, with copper concentrations ranging from 1.6% to 3.8%^25^. Base metal sulfides occurring in metavolcanics formed due to hydrothermal activity linked to volcanism in a subduction zone setting^26^. Khalid and Oweiss^27^ indicated mineralization associated with porphyritic albitites and hydrothermal breccias exhibits features comparable to porphyry copper, magmatic-hydrothermal, and low-sulfidation epithermal systems. The gold and molybdenum concentrations in the Samra area’s copper-bearing shear zones suggest the potential for Cu-Au or Cu-Mo porphyry mineralization^28^.

A comprehensive integration of multi-sensor datasets, comprising Landsat-8, ASTER, Sentinel-1, and the ASTER Digital Elevation Model (DEM), in conjunction with field investigations, petrographical and geochemical analyses, has been employed to pinpoint high-potential zones for hydrothermal ore mineralizations. This multi-disciplinary approach enables high-resolution lithological discrimination, enhances structural interpretation, and provides insights into the tectonic framework of the Samra region, ultimately guiding the delineation of priority targets for copper exploration in southeastern Sinai.

### Geology setting

In southeastern Sinai, the Neoproterozoic succession of metamorphosed sedimentary and volcanic rocks is regionally classified into four principal units: the Malhaq Formation, Um Zariq Formation, Heib Formation, and Tarr Complex^29–31^. Wadi Samra area (Fig. 1b) is part of the Kid metamorphic belt and is occupied by the Tarr complex.

**Fig. 2:**
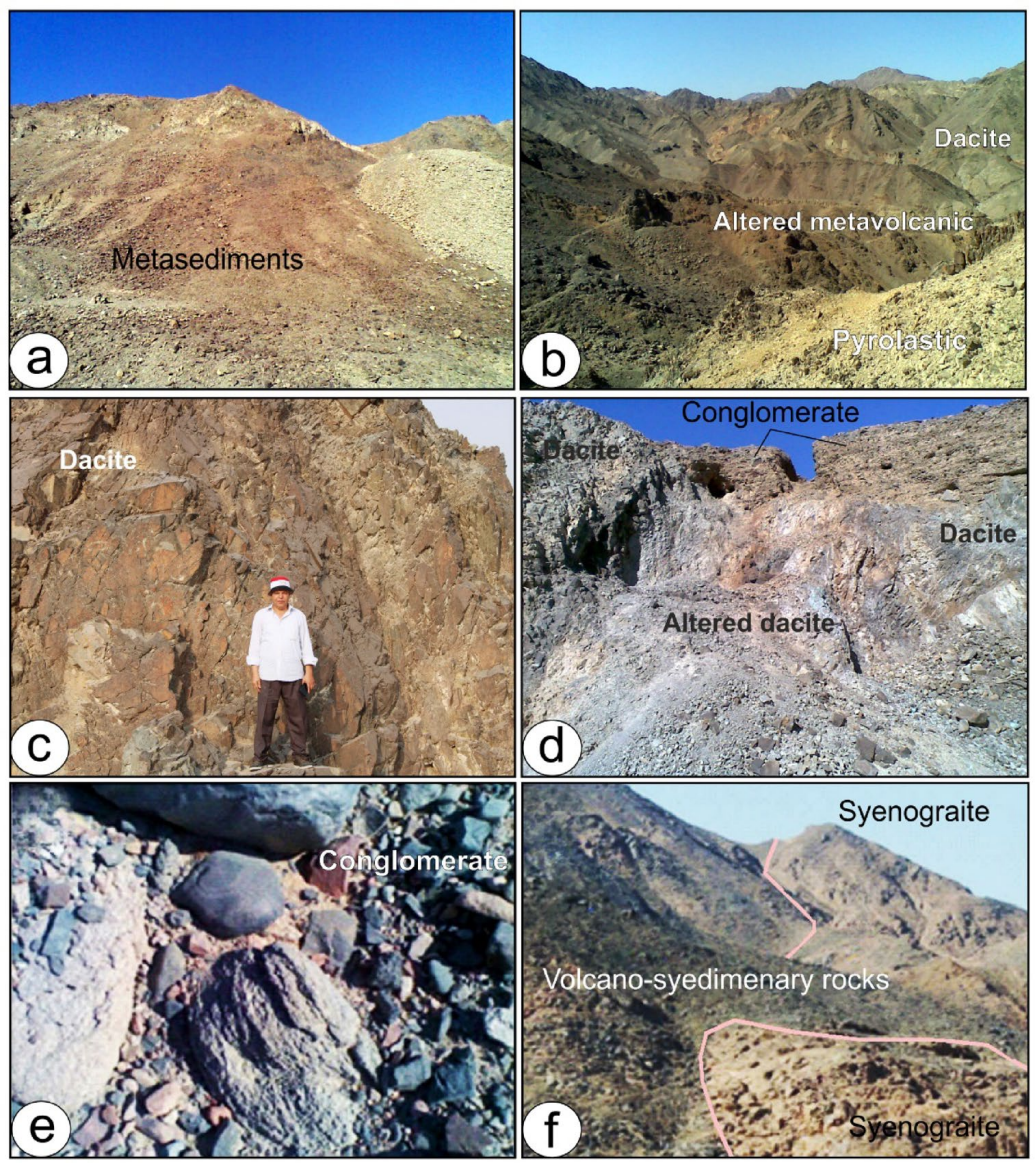
(**a**) Metasediments in southwestern part of Samra area, (**b**) Metavolcanics alteration between lava sheet and pyrolcatics, (**c**) Massive dacite lava sheet, Wadi Kid area, (**d**) Altered dacite overlined by conglomerate, (**e**) NE-trending Kid Conglomerates belt (**f**) Post tectonic granites with rugged topography. intruding the volcano-sediments rocks.

The lithologic diversity of the Tarr Complex reflects a complex volcanic-sedimentary history, characterized by intermediate to felsic volcanic activity and subsequent deformation. It includes metamorphosed lava flows, pyroclastics (ignimbrites, volcanic breccias, and tuffs), and intercalated fine-grained sediments such as tuffaceous and carbonate-rich mudstones, some exhibiting concretionary structures. Ductile deformation has transformed parts of the metasedimentary sequence into schistose units, particularly along shear zones (Fig. 2a). These rocks are intruded by syntectonic granites (older granites), post-tectonic granites (younger granites), and post-granitic dykes.

In the Samra area, volcanic rocks, correlating with the Eastern Desert’s Dokhan volcanics, extend over the southern and central sectors. These volcanic rocks have undergone low-grade metamorphism and include both lava flows and pyroclastics (Fig. 2b). The lava flows are dominated by porphyritic rhyolites, rhyodacites, dacites, and andesites, with less common andesitic basalts (Fig. 2c). The pyroclastic rocks are mainly represented by tuffs, ash-flow welded tuffs, laminated tuffs and less common lapilli-tuff and agglomerate, these pyroclastic rocks are mostly rhyolites–dacite in composition. These volcanic rocks reveal a calc-alkaline affinity, indicating a volcanic arc setting related to subduction processes^17^.

A thick succession of Kid Conglomerates, reaching up to 800 m in thickness, defines a prominent NE-trending lithostructural belt that traverses the study area (Fig. 2d). It shows crude graded bedding with coarse-grained sandstone intervals, polymictic consists mainly of cobble and boulder of metavolcanic and metasedimentary clasts (≈ 1 m length) with some granitoid clasts, set in finer-grained tuffeceous matrix or muddy sand matrix (Fig. 2e). Metavolcanic clasts are derived mainly from the silicic volcanic rocks.

The younger granites are cropping up as rugged topography with moderate to high relief, intruding the older rocks with sharp contacts and in turn invaded by post-granitic dykes (Fig. 2f). These rocks are fine to medium-grained, red to reddish pink in color, represented by monzogranite and syenogranite. The younger granites are exfoliated and weathered. Albitite in an isolated mass (≈ 1.2 km^2^) intrudes the country rocks with a sharp intrusive contact along Wadi Tarr. Smaller albitite bodies occur in the Wadi Ghorabi El-Hatimyia and Wadi Khashm El-Fakh regions.

Field observations indicate that the albitite intrusions are extensively jointed^32^ and exhibit hydrothermal alteration along their margins, including brecciation, carbonatization, and prehnite-bearing zones. The albitite in the smaller intrusive bodies displays a white to light-gray color, medium- to coarse-grained texture, and porphyritic fabric. In the study area, copper and sulfide mineralizations are mainly hosted in metavolcano-sedimentary rocks and intruded by old granites.

## Materials and methods

### Multi-sensor dataset

The multispectral dataset was categorized into free-cloud terrain corrected optical, radar, and Digital Elevation Model (DEM) data. Level-1T scenes from Landsat-8 and ASTER, acquired from the USGS Earth Explorer (http://earthexplorer.usgs.gov) and NASA Earthdata Search (https://search.earthdata.nasa.gov), respectively, were employed in the identification and spatial delineation of lithological contacts, structural lineaments, and hydrothermally altered zones associated with copper mineralization. In contrast, the radar data, represented by Sentinel-1 A obtained from https://search.asf.alaska.edu, along with DEM data from the NASA Earth Data Center, were employed for lineament extraction and creating a hillshade map. The complete remotely sensed data underwent processing through a suite of software packages (e.g., Envi 5.3, ArcMap 10.8, PCIgeomatica 2016, RockWork 2016, CorelDRAW 64-bit), which were stacked and subsetted to delineate the study area. Furthermore, these data were geometrically corrected using the WGS-84 UTM Zone 36 N coordinate system.

Landsat-8 has eleven spectral bands, seven designed to capture reflected solar radiation in the visible, near-infrared (VNIR), and shortwave infrared (SWIR) regions of the electromagnetic spectrum. Band 9 of Landsat-8 is designed explicitly for cirrus cloud detection, while Bands 10 and 11 (TIRS) capture thermal infrared radiation for surface temperature analysis. Band 1 (ultra-blue) is sensitive to coastal and atmospheric aerosol features. The spatial resolution varies across Landsat-8 bands: bands 1–7 and 9 have a 30 m resolution, the panchromatic band (band 8) offers 15 m, and the thermal infrared bands (TIRS 10 and 11) are resampled to 90 mThe Advanced Spaceborne Thermal Emission and Reflection Radiometer (ASTER) comprises 14 spectral bands distributed across three regions: three visible and near-infrared (VNIR) bands at 15 m resolution, six shortwave infrared (SWIR) bands at 30 m, and five thermal infrared (TIR) bands at 90 m spatial resolution. The European Space Agency’s Sentinel-1 satellite carries a C-band synthetic aperture radar (C-SAR) system, operating in interferometric wide-swath (IW) mode with dual polarization (VV/HH and VH/HV). This configuration provides a spatial resolution of 5 m × 20 m.

### Preprocessing and processing of satellite data

The processing of multisensory data occurs in two main stages: preprocessing and processing. The IARR (Internal Average Relative Reflection) method was used for atmospheric correction of Landsat-8 and ASTER data, leveraging the assumption of uniform average reflectance across the scene to normalize pixel values. This correction is critical for accurate spectral analysis, especially in mineral mapping applications where subtle absorption features must be preserved in the VNIR and SWIR regions. It was applied before image classification, band ratioing, and SAM-based mineral identification. In the case of the S1A radar data, the enhanced Lee filter was employed across both VH and VV polarizations to mitigate speckle while ensuring the retention of texture information. Furthermore, a hill-shade model was generated from a Digital Elevation Model (DEM) using the spatial analysis tools in ArcMap. This derived product is a critical base layer for visualizing the spatial distribution of structural lineaments and hydrothermally altered zones.

The processing of satellite data employed in this study is categorized into two principal groups: image enhancement techniques for lithological and structural visualization, and targeted methods for alteration mineral detection and classification. This structured approach ensures a logical workflow from general geological interpretation to specific mineralogical identification.

Image enhancement techniques were applied to improve the visual discriminability of rock units and structural features. False Color Composites (FCC) were generated by assigning specific spectral bands to the red, green, and blue display channels, producing tonal color variations that emphasize the spectral responses of different lithologies and highlight structural lineaments. These composites were further refined using a Decorrelation Stretch (DS) technique, which better enhances color contrast by modifying the principal component space to separate the spectral signatures of different surface materials. The Gram-Schmidt pan-sharpening algorithm was applied to Landsat-8 imagery to achieve higher spatial precision, resampling the multispectral data to a 10-meter resolution.

A synergistic approach was applied to Sentinel-1 A radar data for enhanced structural mapping. A VH + VV intensity composite was generated and combined with the individual VH and VV polarization channels into a three-band dataset. Principal Component Analysis (PCA) was then performed on this dataset, and the resulting components (PC1, PC2, PC3) were analyzed to accentuate linear structural features.

Targeted analytical methods were employed to identify and map minerals associated with hydrothermal alteration directly. Band ratioing was used to calculate the ratio between the reflectance values of two spectral bands, generating grayscale images that emphasize specific mineral absorption features. Using Landsat-8 data, the band ratios 6/4, 6/5, and 7/6 were utilized to highlight ferrous iron oxides (Fe²⁺), ferric oxides (Fe³⁺), and hydroxyl-bearing alterations, respectively. Furthermore, ASTER band ratios 2/1 and 4/2 were applied to delineate the spatial extent of ferrous and ferric iron oxides. These ratios serve as direct proxies for mineral groups including iron oxides and hydroxides and Al/Fe-OH, Mg-Fe-OH, and Si-OH phyllosilicates. Complementing the ratio methods, the Spectral Angle Mapper (SAM) algorithm was applied to the nine ASTER bands for a pixel-based classification. This spectral matching procedure quantifies the similarity between image pixels and reference spectra by computing the angle between their spectral vectors. It enables the systematic detection and mapping of key alteration minerals across the study area.

### Field work

Field investigations were guided by Landsat imagery and 1:50,000-scale geological maps. For subsequent laboratory analysis, representative samples were gathered from ore-bearing rocks, hydrothermal alteration zones, and the related copper and sulfide ores within the examined region.

### Laboratory work

#### Petrographic and mineralogical investigation

Petrographic investigations were performed on 15 polished thin sections from representative rock and ore samples at the Central Laboratory, Assiut University. A Polarizing microscope with both transmitted and reflected light was used to accomplish the petrographic and mineralogical examinations. X-ray diffraction (XRD) analysis was employed to identify the mineralogical composition, utilizing a Philips powder diffractometer fitted with an automated slit system.

Operating conditions for the diffractometer were set at 45 kV and 35 mA, employing Ni-filtered CuKα radiation. Random powder slides were prepared for general mineralogical identification, and semi-quantitative analyses were carried out according to a modified procedure of Schultz^33^. The XRD analyses were performed at the Physics Department Laboratory, Faculty of Science, Sohag University, Egypt.

#### Chemical analysis

Thirty-two representative samples were collected from volcano-sedimentary rocks and the intruded granites in the studied Samra area in the southern Sinai which were chemically analyzed. Major and trace element contents were determined using a Philips X-ray fluorescence spectrometer (model PW/2404) equipped with a Rh radiation tube, with results reported in wt% and ppm. Detection limits ranged from 0.001 to 0.03% for major oxides and 0.01–0.5 ppm for trace elements. Loss on ignition (L.O.I.) was measured by heating 0.5 g of powdered samples at 1000 °C overnight. A total of 119 ore samples from various mineralized sites were analyzed for Cu, Zn, Pb, Au, and Ag at the Central Laboratories of the Geological Survey of Egypt.

## Results

### Remote sensing data

#### Differentiation of lithological units

**Fig. 3:**
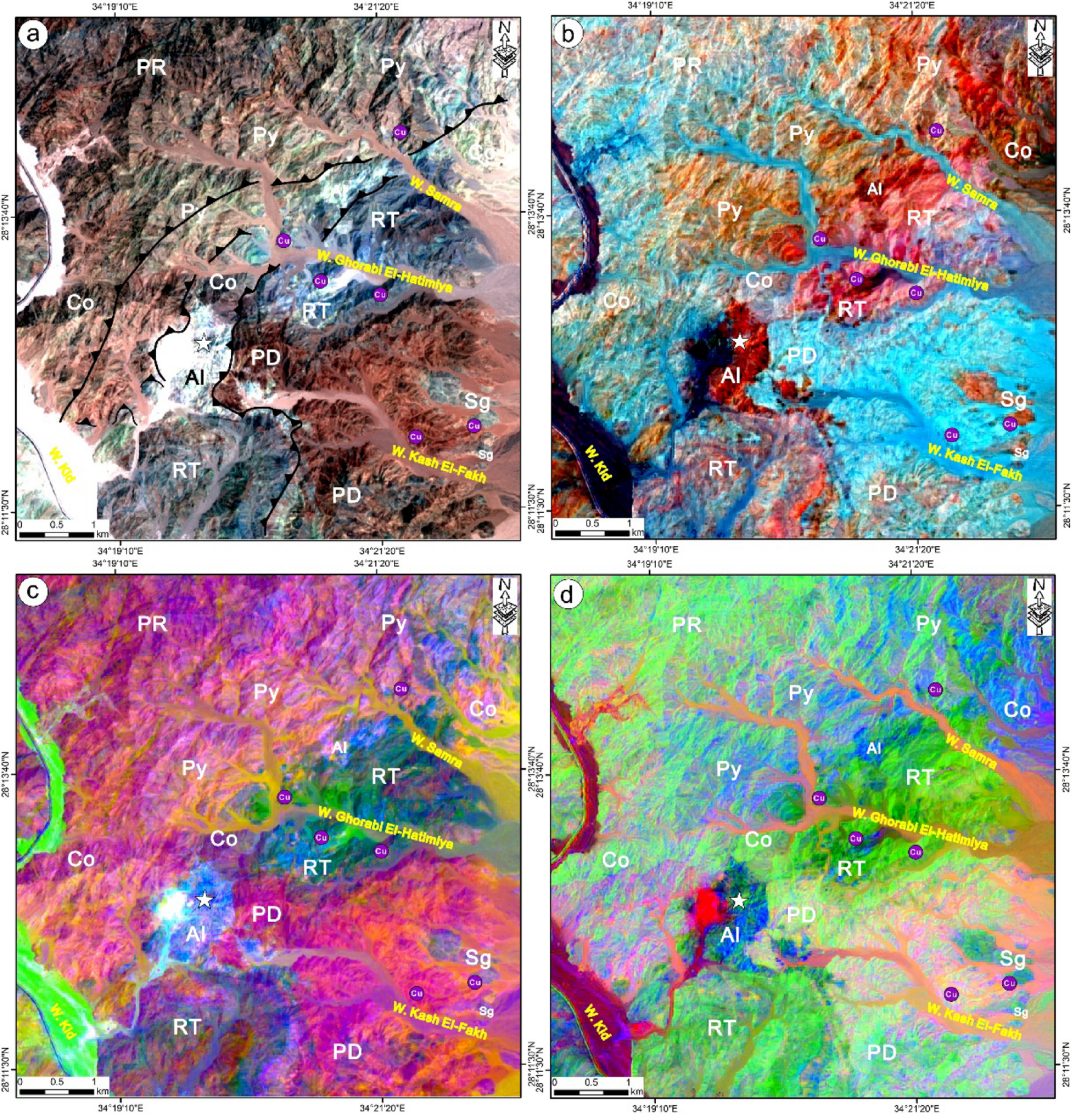
Lithological differentiation utilizing Landsat-8, (**a**) FCC 567, (**b**) BR 1/3 1/7 3/6, (**c**) MNF 234 and (**d**) PC 213 in RGB. Rock abbreviations: porphyritic rhyolite-PR; porphyritic dacite-PD; pyroclastics-Py; rhyodacite tuffs- RT; conglomerate-Co; syenogranite-Sg and albitite- Al. These figures were created and processed by ENVI v. 5.6.2. software: https://www.l3harrisgeospatial.com/Software-Technology/ENVI), which is mainly utilized for image processing, and ArcGIS Desktop 10.8. (https://www.esri.com/en-us/arcgis/products/arcgis-desktop/overview/).

The selected enhanced color composites, including FCC 567, BR 1/3 1/7 3/6, MNF 234, and PC 213 in RGB of Landsat-8, were helpful to highlight the lithological contacts and differentiate among the variable rock units along the area between W. Samra and W. Kid, along with the tracing of the main valleys and the major fault contacts. FCC 567-RGB (Fig. 3a) highlighted the sharp contact between the rock units. It marked each unit with a unique tone as follows: porphyritic rhyolite (PR), light brown; porphyritic dacite (PD), reddish brown; pyroclastics (Py), brownish green; rhyodacite (RT), blue to brownish blue; conglomerate (Co), shiny white-green to green-brown; syenogranite (Sg), bluish grey; and albitite (Al), shiny bluish white.

The volcanic sequence of the presented area was more distinguishable in the BR 1/3 1/7 3/6-RGB (Fig. 3b), where porphyritic rhyolite (PR), porphyritic dacite (PD), pyroclastics (Py), and rhyodacite (RT) appeared in orange-cyan, cyan, light orange, and reddish cyan, respectively. At the same time, the albitite, syenogranite (Sg), and conglomerate (Co) were marked by deep red, dark orange, and pinkish white to orange-white, respectively. The rhyodacite (RT) and porphyritic dacite (PD), as the primary source of porphyritic copper in the present area, were more detectable by their bluish green and deep purple-orange colors in the MNF 234-RGB (Fig. 3c). Moreover, the albitite (Al) quarry was featured in the same composites by its shiny blue to pinkish blue tones. In PC 213-RGB (Fig. 3d), the volcanic sequence featured green, pinkish, bluish, and yellowish green tones, while the albitite (Al) was dark blue.

#### Alteration and iron zones

The methodology for discriminating lithological units and alteration zones was grounded in mineral reflectance spectroscopy, targeting diagnostic absorption features of iron oxides, hydroxyl-bearing minerals, and carbonates. This theoretical framework was statistically validated through Principal Component Analysis (PCA), with the eigenvector loadings (Table 1) quantitatively identifying the paramount contribution of specific spectral bands—notably Bands 5, 6, and 7to the principal components governing spectral variance. Informed by this analysis, a suite of targeted band ratios (Landsat-8 6/4, 6/5, 7/6) was deployed to isolate ferric iron, ferrous iron, and hydroxyl-bearing mineral signatures, respectively. This synergistic approach, which integrates spectroscopic theory with empirical data reduction, ensured a robust and statistically validated capability for mineralogical differentiation, the results of which were subsequently verified through field and geochemical analysis.

**Table 1:** The eigenvector loadings of Landsat-8.

Eigenvector	Band 1	Band 2	Band 3	Band 4	Band 5	Band 6	Band 7
Band 1	– 0.179661	– 0.206011	– 0.280430	– 0.360367	– 0.417157	– 0.539769	– 0.501403
Band 2	0.149076	0.264718	0.424752	0.434299	0.309143	– 0.421962	– 0.514829
Band 3	0.284789	0.367978	0.355779	– 0.070446	– 0.570486	– 0.350987	0.450888
Band 4	0.438752	0.283232	0.041346	– 0.199434	– 0.306403	0.572136	– 0.514365
Band 5	– 0.612256	– 0.198465	0.531584	0.221218	– 0.400201	0.280407	– 0.124282
Band 6	– 0.314470	0.341084	0.350667	– 0.722839	0.373097	– 0.009253	– 0.004516
Band 7	0.445130	– 0.718421	0.455091	– 0.255637	0.108221	– 0.036772	0.014431

**Fig. 4:**
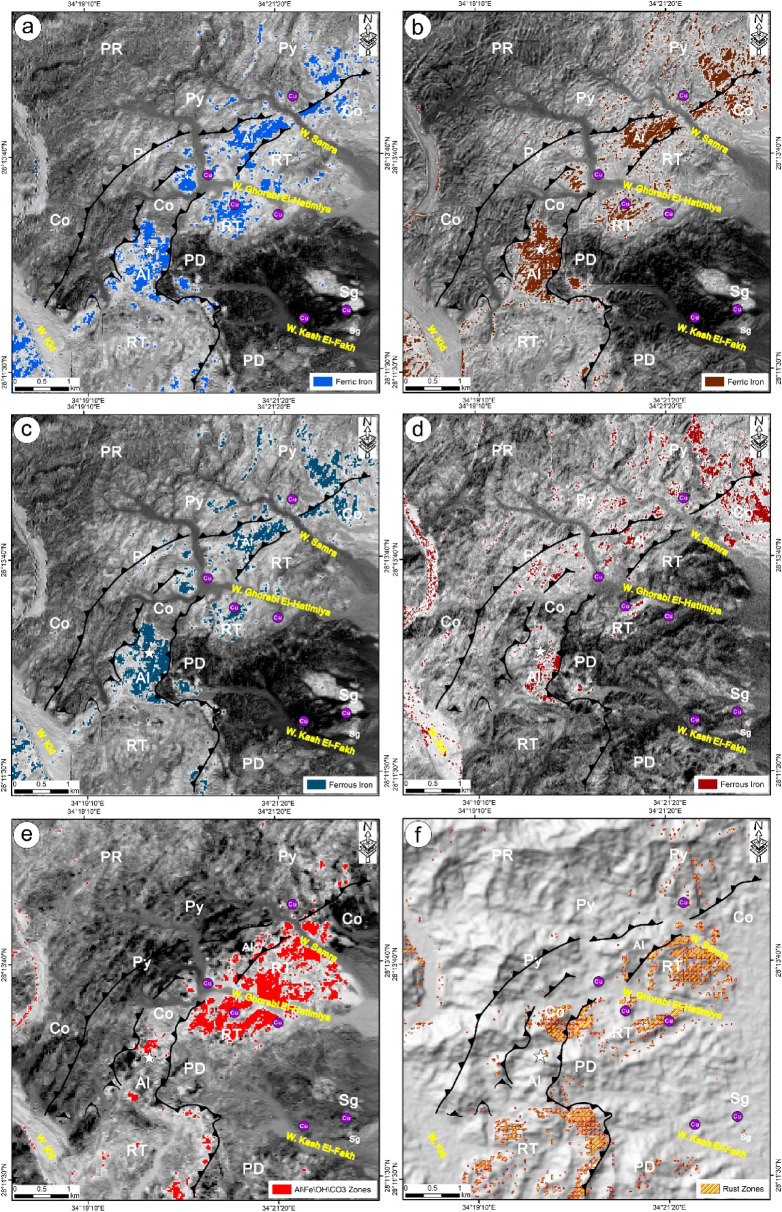
Alteration and Iron mapping via both landsat-8 (**a**) Ferric iron, (**b**) Ferrous iron, (**c**) Al, Fe, OH, CO_3_ zones; and ASTER (**d**) Ferric iron, (**e**) Ferrous iron, (**f**) Rust zones. Rock abbreviations see Fig. 3. These figures were created and processed by ENVI v. 5.6.2. software: https://www.l3harrisgeospatial.com/Software-Technology/ENVI), which is mainly utilized for image processing, and ArcGIS Desktop 10.8. (https://www.esri.com/en-us/arcgis/products/arcgis-desktop/overview/).

**Fig. 5:**
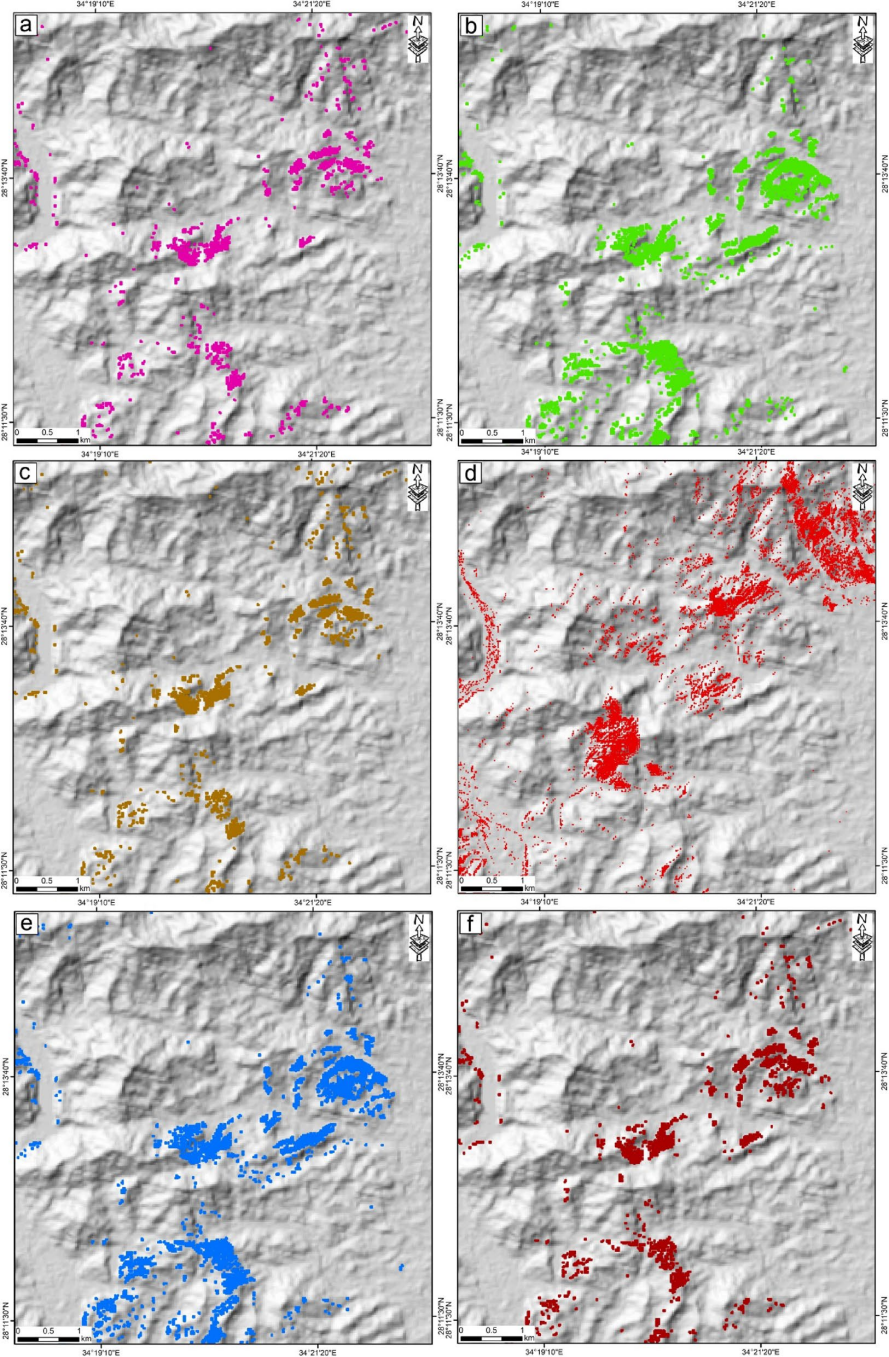
Spatial distribution of the allocated alteration minerals; (**a**) Muscovite, (**b**) Chlorite, (**c**) Biotite, (**d**) Ferric and Ferrous irons, (**e**) Epidote and (**f**) Jarosite.

Using Landsat-8 spectral bands, grayscale ratios of 6/4, 6/5, and 7/6, which were successfully applied in numerous peer-reviewed geological remote sensing studies^13,14,20,24,34–36^ were employed to highlight ferrous iron oxides, ferric oxides, and hydroxyl-bearing alterations, respectively. These indices indicate mineral groups including Fe³⁺, Fe²⁺, Al/Fe-OH, Mg-Fe-OH, and Si-OH (Figs. 4a, b, and c). Furthermore, the band ratios 2/1 and 4/2 derived from ASTER imagery were utilized to delineate the occurrence and spatial extent of ferrous and ferric iron oxides within the investigated region (Figs. 4d and e). In the context of mapping hydrothermal alteration minerals and along with the utilized BRs of Landsat-8 and ASTER linked to iron and rust zones as a key alteration zone within the copper belts, the spectral properties of hematite, jarosite, biotite, muscovite, chlorite and epidote were analyzed for identification through the SAM technique applied on ASTER data to allocate the rust zones in the examined area (Figs. 4f and 5a-f). The surface distributions of the rust zone minerals were assigned using the spectral library provided by USGS and by moving the rule threshold while applying the SAM method provided by Envi to a value equal 0.650. The entire detected rust minerals were merged as one shape file using ArcMap software to allocate the rust zones associated with the copper ore areas between W. Samra and W. Kid (Fig. 4f).

#### Lineament extraction

For this analysis, Sentinel-1 A imagery was geometrically refined and subsequently filtered using the adaptive enhanced Lee method, effectively minimizing speckle effects and improving the delineation of structural features^37^. Processing of Sentinel-1 A SAR data included PCA-based statistical analysis, resulting in the extraction of PC1, PC2, and PC3 images that enhanced the recognition of critical structural patterns and lineaments.

**Fig. 6:**
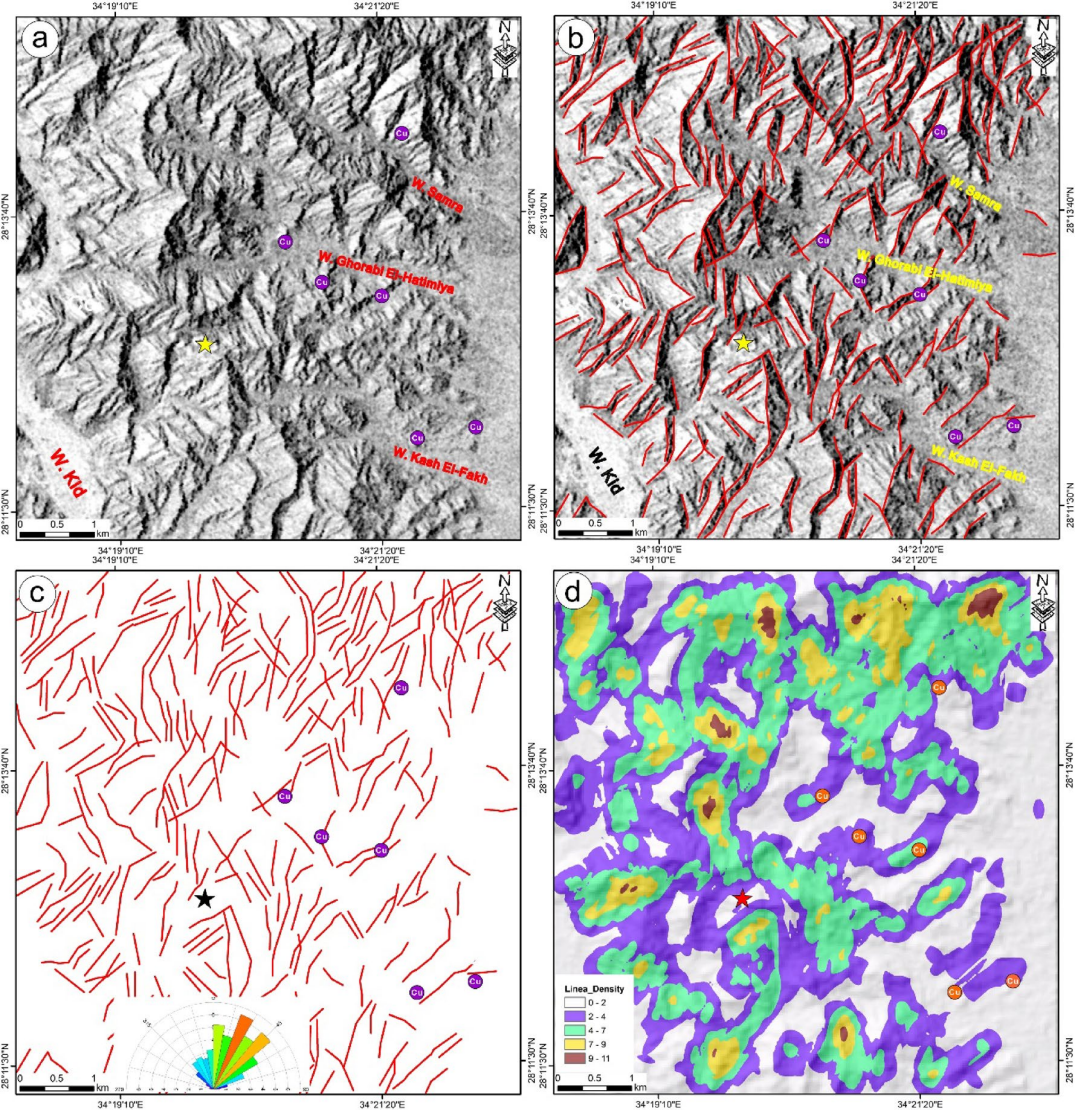
Lineament extraction via S1A radar data; (**a**) PC1, (**b**) PC1 and sptatial distribution of extracted lineaments, (**c**) Lineament map and azimuth diagram, (**d**) Lineament Density map. Rock abbreviations see Fig. 2. These figures were created and processed by ENVI v. 5.6.2. software: https://www.l3harrisgeospatial.com/Software-Technology/ENVI), which is mainly utilized for image processing, and ArcGIS Desktop 10.8. (https://www.esri.com/en-us/arcgis/products/arcgis-desktop/overview/).

The first principal component (PC1; Fig. 6a) of Sentinel-1 A data represents the key structural information for the investigated region. Using the LINE algorithm available in PCI Geomatica, this component was analyzed to automatically delineate lineaments and linear geological features (Figs. 6b and c). This principal component was subsequently utilized for the automatic extraction of lineaments, highlighting their spatial distribution and azimuth frequency within the study region (Fig. 6c). Moreover, the extracted lineaments were processed using ArcMap software to produce a density map, illustrating the distribution of lineament concentrations across different rock units (Fig. 3d).

The azimuth frequency diagram (Fig. 6c) shows that lineaments predominantly trend NE, followed by ENE, NNE, and NW orientations in descending frequency. Moreover, the lineament map revealed that the copper occurrences alongside the albitite quarry are aligned to the linear structures striking mainly NE to ENE (Figs. 6b and c). The density map reveals that the study region is characterized by moderately lineament concentrations throughout the volcanic sequence and syenogranite rocks (Fig. 6d).

### Structures

**Fig. 7:**
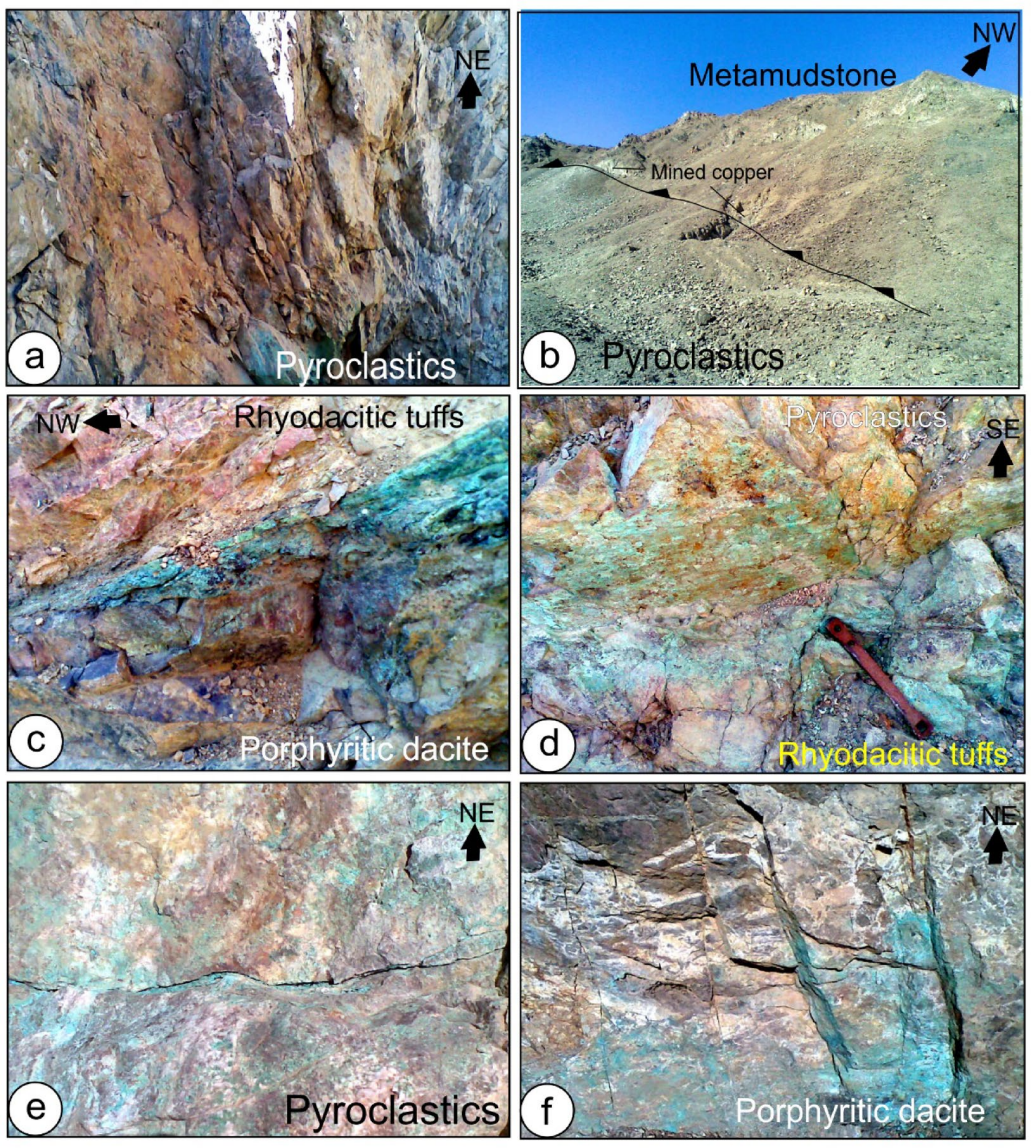
(**a**) Mined copper along the NW-dipping thrust contact between metamudstone and pyroclastics, (**b**) Subvertical shear zone in altered pyroclastics, (**c**) Copper mineralization along the thrust contact between rhyodacitic tuffs and porphyritic dacite, (**d**) Copper mineralization and small quartz veins along the thrust contact between pyroclastics and rhyodacitic tuffs, (**e**) slightly folded copper mineralization in pyroclastics, (**f**) Copper mineralization in faulted and brecciated dacite.

Within the Tarr Complex, early structural work attributes D1 to a regional folding episode characterized by tight to isoclinal geometries, steep-to-overturned limbs, and fold axes plunging preferentially toward the NE or SW. The geometry of D1 folds offers a consistent interpretation for the regionally steepened stratification observed across the study area. This interpretation accounts for the consistently steep bedding dips observed in the area^37–42^. According to Fowler et al.,^43^, the Tarr Complex in the Wadi Samra region underwent three distinct phases of deformation: (i) D1, which led to the formation of northeast-trending steep to overturned strata (Fig. 7a); (ii) D2, characterized by recumbent folding, marked by sub-horizontal S2 cleavage, and accompanying low-angle thrust oriented southeastward (Fig. 7b, c and d); and (iii) D3, which denotes an upright macroscopic folding event trending west-northwest to east-southeast (Fig. 7e).

The Tarr Complex comprises fine-grained pyroclastics, clastics, volcanic materials, and ignimbrites. Albitite and carbonatite intrusions in the area are likewise well established. The relationships among rhyodacitic tuffs, pyroclastics, albitite, and porphyritic dacite are influenced by thrust faults that dip to the northwest. The metavolcanic succession of the Tarr Complex exhibits only limited development of folds. The occurrence of asymmetric folds is confined mainly to discrete shear zones within the Tarr Complex, indicating their origin as shear-induced structures. Additional folding has resulted in the buckling of shear zones, typically producing extensive, open, rounded folds. While uniformly spaced folds are rare, they can be observed in specific sequences of metamudstone within the Tarr Complex^43^. The ignimbrites in this area exhibit excellent eutaxitic textures and retain pumice fragments. The finer-grained tuffs show bedding laminations and graded bedding. In the Kid Conglomerates, the bedding features alternating pebbly bands and sandy strata^43^. In outcrops of pyroclastics, bedding is discernible as interlayered tuffs and bands with higher carbonate content. The northern section of the Wadi Samra region features a northwest-dipping thrust zone that has been folded around axial planes trending from west-northwest to east-southeast. This thrust boundary is associated with a significant development of S2 foliation, likely indicative of a D2 thrust structure^38,43^. One can find dark, fine-grained metamorphosed mudstones and graded metatuffs in the northeastern part of Wadi Samra. At the same time, adjacent to these units in the southeast, volcanic breccias are exposed. The geological units in the Wadi Samra area are characterized by steeply dipping to overturned strata, with a dominant NE–SW structural trend and a consistent younging direction toward the southeast. In the northern Wadi Kid area, dently inclined foliations slightly modify the steep bedding, revealing subtle cleavage, recumbent open folds trending NE-SW, and shallow-dipping shears, corresponding to the D2 structures^43^. F2 folds are extensively present in the southwestern area^38^.

In the Samra region, copper mineralization is linked to hydrothermal alteration processes. These alterations result in areas abundant in copper, gold, and silver, with notable mineralization in conjunction with argillic, phyllic, and propylitic alterations. The mineralization is frequently associated with fault zones and structural characteristics that promote the flow of hydrothermal fluids. The existence of shear zones and faulting (Fig. 7f) has been instrumental in the concentration of copper mineralization. These geological structures facilitate the movement of hydrothermal fluids, which are vital for accumulating copper minerals.

### Petrography

**Fig. 8:**
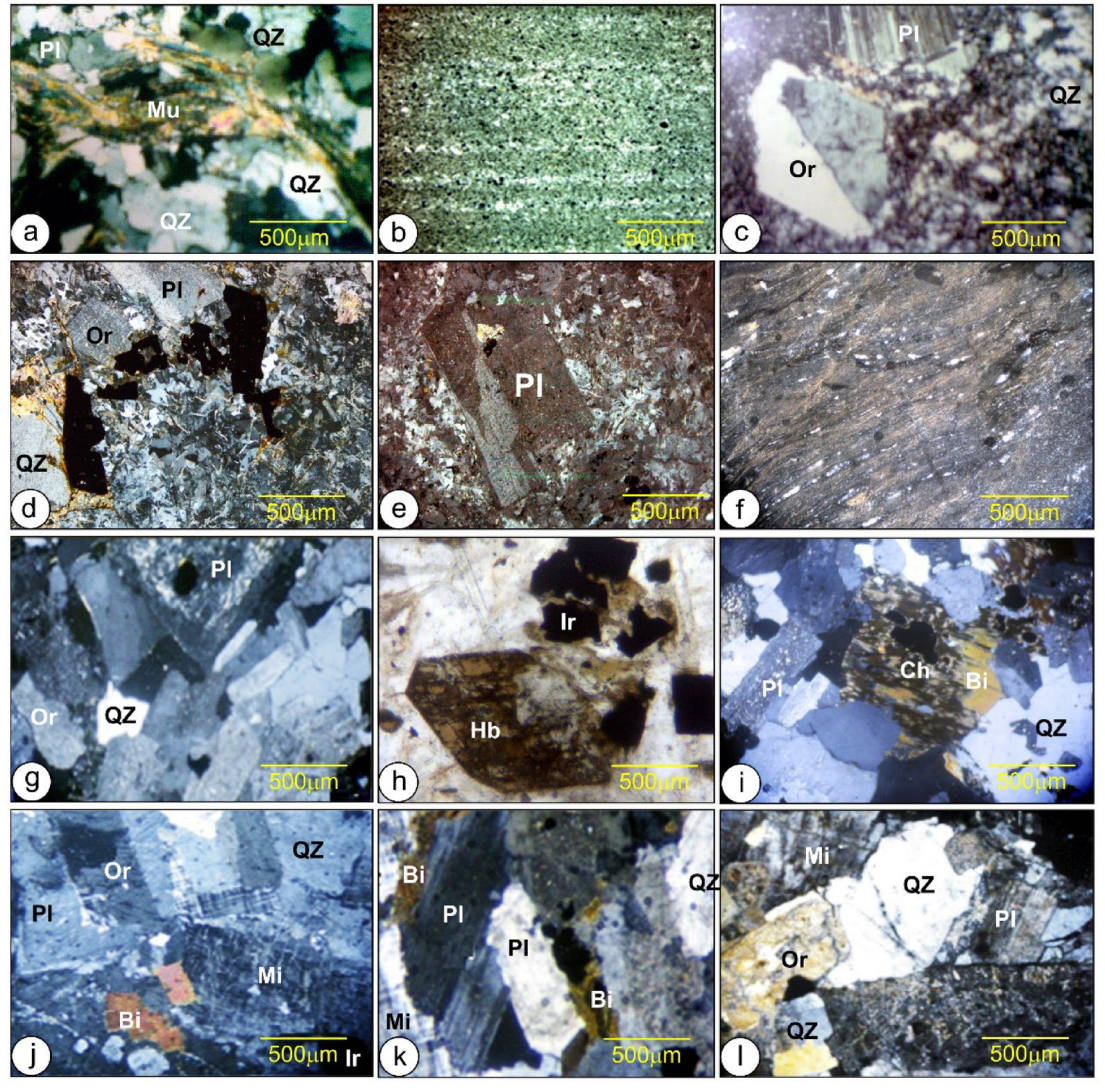
(**a**) Medium-to-coarse-grained arenite that is mainly made up of quartz, plagioclase with muscovite, (**b**) Metamudstone built up of clay- and silt-sized quartz, clay minerals and lot of chlorite, (**c**) Porphyritic metarhyolites composed of phenocrysts of subhedral K-feldspar, and plagioclase and quartz set in an aphanitic groundmass mainly composed of quartz, K-feldspar flakes of biotite and chlorite, (**d**) Metadacites composed of plagioclase phenocrysts less amount of quartz and alkali feldspar, set in microcrystalline groundmass composed of plagioclase lathes, quartz and flakey biotite/chlorite and opaques iron oxides, (**e**) Metaandesites are mainly composed of altered plagioclase phenocrysts are embedded in groundmass consists mainly of plagioclase laths with subordinate quartz in addition to chlorite and opaques minerals mainly iron oxide, (**f**) Ignimbrite composed of altered glass shards and pumaceous fragments displaying flow texture, (**g**) Quartz-diorite composed of, plagioclase, mafic minerals and quartz, with minor alkali feldspars; (**h**) Quartz-diorite show mafic minerals (hornblende) altered into iron oxide (magnetite), (**i**) Granodiorite consist of plagioclase and quartz with subordinate alkali feldspars and biotite, (**j**) Biotite syenogranite consists of quartz, alkali feldspars, plagioclase and biotite, (**k**) Syenogranite consists of quartz, alkali feldspars, plagioclase and biotite, note the replacement of biotite mineral by iron oxide, (**l**) Alkali granite composed of perthite (microcline and/or orthoclase), quartz, plagioclase. Pl; Plagioclase, Mi; Microcline, Qz; Quartz, Hb; Hornblende, Ch; Chlorite; Bi; Biotite, Ep; Epidote, Or; Orthoclase, Mu; Muscovite.

Metaconglomerates, metamudstones, and metasandstones represent metasediments along Wadi Samra and downstream of Wadi Kid. These sediments show lower-grade metamorphism of schist-grade conditions, equivalent to the Tarr Formation and El Beida sediments. Metaconglomerates are poorly sorted, coarse-grained, and arranged into beds that range in thickness from 2 to 10 m. They are mainly boulder to cobble-sized and sub-angular to sub-rounded in shape. Metaconglomerates overlie metasandstone with an erosional surface and represents tabular, sheet-like beds that are 5–10 m thick. This rock is texturally immature and poorly sorted, with a medium- to coarse-grained fabric. Lithologically, it is classified as a metamorphosed subarkosic arenite, dominated by quartz and plagioclase feldspars, accompanied by minor orthoclase and lithic fragments derived from sedimentary and volcanic sources (Fig. 8a). Metamudstones are represented by 1.5 m thick beds that build to a significant thickness of several tens of meters. They are constructed of clay- and silt-sized quartz, clay minerals, and many chlorites (Fig. 8b).

Metavolcanic rocks are represented by acidic, intermediate, and less common basic lava flows and volcaniclastic rocks. Lava flows include metarhyolites, metarhyodacites, metadacites, and matandesites. The porphyritic type, with a lesser aphyric type, dominates metarhyolites. The mineral assemblage of porphyritic metarhyolites consists of subhedral K-feldspar, quartz, and plagioclase phenocrysts, accompanied by lesser biotite and hornblende. These are hosted within an aphanitic groundmass made up primarily of quartz, K-feldspar, plagioclase, biotite, chlorite, and opaque minerals (Fig. 8c). Metadacites are characterized by a porphyritic texture defined by abundant plagioclase phenocrysts with subordinate quartz, alkali feldspar, biotite, and hornblende. These phenocrysts are enclosed in a fine-grained groundmass of plagioclase laths, quartz, mafic phases (biotite, chlorite), and scattered opaque oxides, predominantly hematite (Fig. 8d).

The metaandesites contain phenocrysts of plagioclase, augite, and hornblende, with plagioclase extensively altered to sericite, chlorite, and calcite. Secondary phases include epidote, while iron oxides and apatite are accessories (Fig. 8e).

Pyroclastics in the southeastern part along Wadi Samra are represented by felsic composition, mainly of rhyolite to dacite in composition. The crystal tuffs are characterized by abundant phenocrysts and fragments embedded in a fine-grained matrix, with sparse lithic fragments derived from surrounding country rocks. Lithic fragments are represented by sub-angular to sub-rounded metadacite/metarhyolite set in tuffaceous fine-grained groundmass consisting of quartz, plagioclase, and dusty material. Ignimbrite comprises altered glass shards and pumaceous fragments displaying flow texture (Fig. 8f).

Syntectonic granitoids, particularly quartz diorite and granodiorite, represent critical intrusive phases in the study area. Quartz diorite is mineralogically dominated by calcic plagioclase, quartz, and amphiboles (hornblende and actinolitic hornblende), with subordinate alkali feldspar. It is characterized by a fine- to medium-grained texture (Figs. 8g and h). Accessory minerals in these granitoid rocks include iron oxides, titanite (sphene), zircon, and apatite, whereas secondary alteration products are dominated by epidote, chlorite, and sericite.

The granodiorites are leucocratic to mesocratic and are mineralogically dominated by plagioclase, quartz, alkali feldspar, and biotite (Fig. 8i), with accessory minerals including iron oxides, mainly magnetite (indicating magnetite-series granitoids). They typically display medium- to coarse-grained textures that range from porphyritic to equigranular–hypidomorphic.

Younger granite petrographically is represented by biotite syenogranite and alkali granite.

The biotite syenogranite is coarse-grained, massive, and pinkish-grey in color, reflecting its high-K, peraluminous composition. Its mineralogy is dominated by quartz, alkali feldspar, plagioclase, and biotite, with accessory phases including iron oxides, zircon, and apatite. Post-magmatic alteration has generated secondary minerals such as sericite, chlorite, and epidote. Porphyritic and myrmekitic fabrics are texturally well developed (Figs. 8j and k). Alkali granite is a coarse-grained, massive rock with a pinkish-grey hue, composed predominantly of perthite, quartz, plagioclase, biotite, and muscovite. Accessory phases are represented by iron oxides, apatite, and zircon, whereas secondary alteration has produced sericite, chlorite, epidote, sphene, and allanite. Texturally, the rock is characterized by hypidiomorphic, perthitic, and myrmekitic fabrics (Fig. 8l).

### Geochemistry

**Fig. 9:**
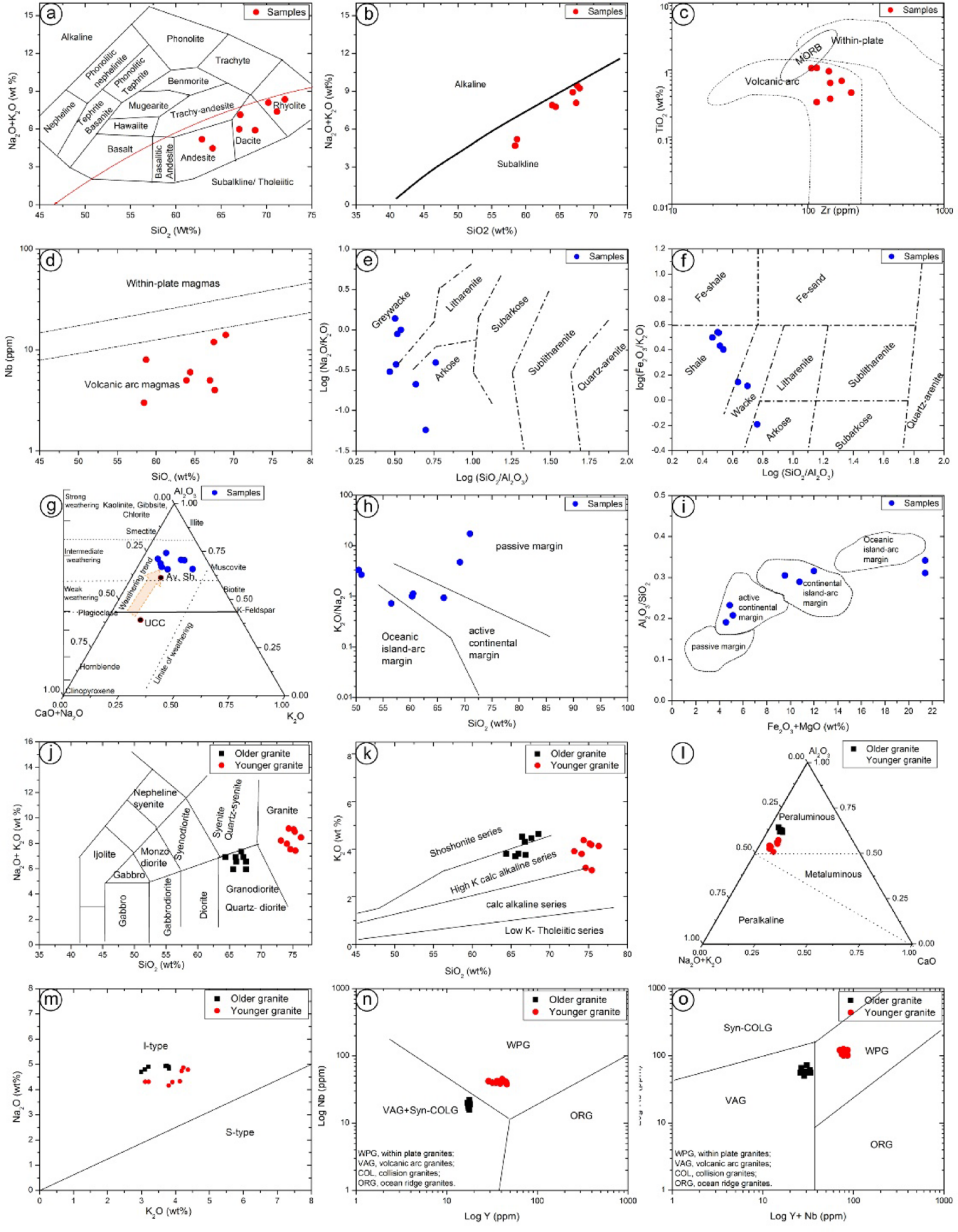
(**a**) Plotting the metavolcanics samples in Na_2_O + K_2_O vs. SiO_2_binary diagram^44^; (**b**) Alkalis-silica diagram^45^ of the metavolcanics; (**c**) Zr vs. TiO_2_ diagram^46^ of the metavolcanics rocks, (**d**) plotting the metavolcanics samples in SiO_2_vs. Nb diagram^47^, (**e**) Classification the studied metasediments according to log (Na_2_O/K_2_O) vs. log (SiO_2_/Al_2_O_3_) diagram^52^, (**f**) Classification the studied metasediments according to log (Fe_2_O_3_/ K_2_O) vs. log (SiO_2_/Al_2_O_3_) diagram^53^, (**g**) Weathering history of the studied metasediments accordingAl_2_O_3_–(CaO + Na_2_O)–K_2_O diagram^49^, (**h**) Log (K_2_O/Na_2_O) vs. SiO_2_ diagram^50^ illustrate the geological tectonic settings of the volcaniclastic metasediments, (**i**) Al_2_O_3_/SiO_2_ vs. (Fe_2_O_3_* + MgO) discrimination diagrams^51^ provide details of geological tectonic settings of the volcaniclastic metasediments. (**j**) Geochemistry of the granitic intrusive rocks at Samra area. (**k**) Total alkalis (Na_2_O + K_2_O) versus SiO_2_, (**l**) K_2_O versus SiO_2_; (m) Al_2_O_3_-CaO-(Na_2_O + K_2_O) diagram, (**n**) K_2_O versus Na_2_O, (**e**) Nb (ppm) versus Y (ppm), (**o**) Nb + Y (ppm) versus Rb (ppm).

The geochemical composition of metavolcanics was investigated (Table 2). Based on the Na_2_O + K_2_O versus SiO_2_ diagram^44^, the metavolcanic samples fall within the fields of andesite, dacite, and rhyolite (Fig. 9a).

**Table 2:** Representative whole rock major oxides (wt%) and trace elements (ppm) for samples from metavolcanics and metasediments.

	Metavolcanics								Metasediments							
Samp.No.	1	2	3	4	5	6	7	8	1	2	3	4	5	6	7	8
SiO_**2**_	58.44	58.71	63.91	64.41	67.44	67.95	66.94	67.55	55.9	61.22	60.57	70.45	70.01	74.81	52.5	51.19
TiO_**2**_	1.11	1.06	1.06	1.07	0.64	0.39	0.37	0.33	0.7	0.75	0.61	0.54	0.43	0.23	0.67	0.91
Al_**2**_**O**_**3**_	16.55	16.55	15.67	15.45	15.32	15.27	15.55	15.11	16.95	16.94	17.35	15.21	15.91	13.16	17.35	15.73
Fe_**2**_**O**_**3**_	5.01	4.99	5.34	5.45	4.68	3.18	4.78	4.15	7.85	6.21	8.18	4.65	3.93	2.51	13.15	15.95
MnO	0.11	0.12	0.12	0.11	0.12	0.13	0.16	0.19	0.11	0.12	0.11	0.12	0.13	0.11	0.15	0.14
MgO	4.57	4.35	1.67	1.47	1.17	0.71	0.38	0.47	3.72	3.78	2.45	1.15	1.13	0.85	4.25	3.45
CaO	5.59	5.17	2.28	2.65	1.32	1.95	1.34	1.45	3.87	2.55	2.57	1.15	1.16	1.25	1.77	1.55
Na_**2**_**O**	3.33	3.37	4.32	4.34	4.35	5.12	4.38	4.83	3.49	2.78	2.36	1.25	1.55	2.15	2.68	2.95
K2O	1.35	1.83	3.58	3.43	3.73	4.13	4.55	4.63	2.57	2.78	2.74	3.35	3.51	3.75	5.15	5.24
P_**2**_**O**_**5**_	1.27	1.27	0.19	0.12	0.14	0.19	0.15	0.19	0.14	0.12	0.11	0.18	0.12	0.12	0.13	0.12
LOI	1.67	1.87	1.79	0.99	0.95	0.93	0.49	0.98	2.89	2.58	2.85	1.95	2.11	1.15	1.95	2.61
Total	99	99.29	99.93	99.49	99.86	99.95	99.09	99.88	99.19	99.83	99.9	100	99.99	100.09	99.75	99.84
Sc	13	12	15	14	13	22	21	22	18	14	18	13	20	22	33	28
V	139	116	164	155	115	191	173	136	195	188	127	149	127	125	273	270
Cr	133	128	165	180	245	180	194	153	127	97	105	87	59	38	49	151
Co	50	51	64	57	67	44	42	57	42	45	41	4	51	53	37	29
Ni	20	18	24	30	39	34	58	43	29	38	37	16	19	18	45	41
Zn	79	82	69	73	75	58	78	77	91	69	75	39	36	46	37	42
Ga	21	22	26	22	29	27	23	27	21	25	20	23	21	24	23	29
Rb	49	52	54	45	63	75	43	53	79	85	99	68	94	59	98	91
Sr	595	545	485	435	305	295	425	581	422	163	281	141	144	156	133	125
Y	17.11	18.01	16,78	18.12	18.15	15.98	14.99	20	21	32	24	31	27	28	23	26
Zr	166	206	106	116	146	246	146	116	120	189	125	182	177	137	184	179
Nb	3	8	5	6	12	23	5	4	9	10	8	7	6	5.	8.	7
Ba	585	625	165	445	815	565	148	307	639	651	789	667	994	997	579	521
Pb	11	13	12	13	11	10.78	11.01	12.12	0.11	0.33	0.53	0.15	0.27	0.18	0.23	0.14
Th	6	10	8	6	8	8	6	5	5	6	7	9	9	12	10	7
U	4	7	10	5	6	4	6	4	3	10	9	7	5	2	8	4

Based on alkali–silica plot^45^, the metavolcanics exhibit subalkaline characteristics (Fig. 9b). On the Zr versus TiO_2_ discrimination diagram^46^ (Fig. 9c), the metavolcanic rocks fall within the volcanic arc field. This interpretation is further corroborated by the SiO_2_ versus Nb plot (Fig. 9d), where most samples also align with the volcanic arc magma domain^47^.

Changing feldspars to clay minerals enriches the altered samples with K_2_O and Al_2_O_3_. As a result, the majority of the minerals in the present samples are clay minerals (Figs. 9e, f, and g), with mica and k-feldspars among the rock-forming minerals^48^, and may be formed due to the action of hydrothermal solutions. The present work employs the (K_2_O/Na_2_O) ratio logarithmic plot versus the SiO_2_ discrimination diagram^50^ to illustrate the geological tectonic settings of the volcaniclastic metasediments (Fig. 9e). Figure 9h indicates that the geochemical data of the volcaniclastic metasediments are mainly clustered within the ACM and OIA tectonic fields. Based on Al₂O₃/SiO₂ versus (Fe₂O₃* + MgO) discrimination diagrams, the samples fall within continental arc and island arc fields, indicating varied sources of detrital material input^51^. Samples in (Fig. 9i) are distributed between the ACM and OIA fields, confirming the multiple sources. This variability reflects a mixed provenance, with detrital contributions from both continental arc (high-K calc-alkaline granitoids) and island arc (volcanic clasts) sources, consistent with the complex tectonic evolution of the Kid Metamorphic Belt^43,54^.

To investigate the magmatic characteristics, the older and younger granites of the Samra area were analyzed for their major and trace element contents (Table 3).

**Table 3:** Representative whole rock major oxides (wt%) and trace elements (ppm) for samples from syn- and post tectonic granites.

Samp. No.	Syn-tectonic granites (Older granites)								Post tectonic granites (Younger granites)							
	1	2	3	4	5	6	7	8	1	2	3	4	5	6	7	8
SiO_**2**_	64	64.1	64.01	63.9	65.73	65.53	65.7	65.6	73.15	74.1	75.42	74.63	74.37	75.34	76.31	75.14
TiO_**2**_	0.3	0.3	0.21	0.24	0.22	0.31	0.21	0.23	0.15	0.16	0.12	0.13	0.13	0.14	0.6	0.09
Al_**2**_**O**_**3**_	17.73	17.14	17.65	17.57	15.66	15.53	15.54	15.57	12.32	12.25	11.76	12.22	12.13	11.19	11.18	11.34
Fe_**2**_**O**_**3**_	2.3	2.5	2.5	2.4	0.55	1.34	1.27	1.3	1.1	1.18	1.6	1.09	0.48	0.15	0.76	0.54
FeO	2.5	2.6	2.5	2.5	2.95	2.5	1.8	2.6	0.8	0.95	0.71	0.89	0.5	1.32	0.3	0.86
MnO	0.1	0.11	0.12	0.11	0.14	0.15	0.12	0.13	0.71	0.11	0.04	0.5	0.1	0.08	0.12	0.1
MgO	1.13	1.3	1.03	1.1	1.16	1.37	1.8	1.5	0.92	0.83	0.7	0.65	0.49	0.33	0.41	0.25
CaO	1.16	1.75	1.1	1.9	1.88	1.39	1.47	1.4	1.85	1.65	1.61	1.62	1.1	1.23	1.23	1.89
Na_**2**_**O**	4.95	4.83	4.9	4.93	4.8	4.7	4.9	4.8	4.3	4.16	4.31	4.31	4.79	4.73	4.33	4.87
K_**2**_**O**	3.75	3.81	3.8	3.7	5.09	4.99	5.2	5.1	3.91	3.8	3.11	3.21	4.37	4.18	4.13	4.23
P_**2**_**O**	0.01	0.02	0.02	0.01	0.01	0.01	0.02	0.02	0.14	0.16	0.11	0.18	0.2	0.09	0.11	0.01
L.O.I.	1.6	1.01	1.69	1.64	1.5	1.7	1.8	1.6	0.52	0.5	0.5	0.5	1.1	1.2	0.5	0.5
Total	99.53	99.47	99.53	100	99.69	99.52	99.83	99.85	99.87	99.85	99.99	99.93	99.76	99.98	99.98	99.82
Zr	266	214	254	247	255	231	251	249	295	326	215	293	257	263	255	293
Y	17.81	17.43	17.51	18.01	16.89	17.23	17.83	17.92	45	39	29	32	36	35	46	41
Sr	774.03	799.21	789.29	769.94	779	737	763	750	114	111	101	105	119	111	113	109
Rb	56	61	59	58	68	57	59	61	121	125	121	118	101	105	114	101
Nb	16	18	17	19	20	19	22	20	41	39	42	40	42	40	38	45
Ba	691	619	632	671	916	912	694	876	250	258	266	256	285	237	244	256
Pb	15	23	21	22	49	61	39	45	49	29	41	50	56	37	66	57
Ga	21	19	22	21	25	19	21	23	21	21	22	23	25	22	21	22
Cu	10	12	10	11	26	25	23	20	25	24	34	22	34	19	25	18
Ni	9	7	8	8	11	12	9	11	10	7	11	7	10	8	7	8
Co	10	9	10	9	10	8	9	8	7	8	9	9	7	9	8	9
Cr	53	61	55	60	61	50	55	65	11	21	21	17	17	16	11	18
V	53	52	58	54	73	73	58	67	14	17	12	15	13	16	12	13
Zn	56	65	51	58	53	77	67	64	51	49	47	44	60	71	67	62

**Table 4:** Cu, Pb and Zn (wt.%) and Au and Ag (ppm) for 119 samples from mineralized zones Continued.

	Latitude	Longitude	Cu %	Pb %	Zn %	Au ppm	Ag ppm
Wadi Khashm El-Fakh area	28°11’46.50"N	34°22’2.11"E	1.44	0.05	0.06	0.11	0.51
	28°11’54.08"N	34°22’4.79"E	1.30	0.15	0.03	0.14	0.39
	28°11’53.12"N	34°21’48.51"E	1.13	0.13	0.12	0.12	0.29
	28°11’54.30"N	34°21’47.84"E	0.8	0.06	0.05	0.16	0.28
	28°11’52.83"N	34°21’47.33"E	0.93	0.01	0.02	0.11	0.37
	28°11’51.77"N	34°21’51.87"E	0.91	0.02	0.02	0.19	0.27
	28°11’54.40"N	34°21’51.04"E	0.91	0.01	0.02	0.2	0.37
	28°11’47.07"N	34°22’0.36"E	0.90	0.01	0.02	0.11	0.30
	28°11’48.79"N	34°22’6.13"E	0.82	0.05	0.05	0.15	0.41
	28°11’47.74"N	34°22’10.24"E	0.82	0.05	0.04	0.13	0.61
	28°11’45.78"N	34°22’3.72"E	0.84	0.02	0.02	0.13	0.81
	28°11’53.52"N	34°22’5.31"E	0.59	0.12	0.17	0.12	0.41
	28°12’1.05"N	34°22’14.12"E	0.57	0.07	0.22	0.16	0.37
	28°12’2.99"N	34°22’10.92"E	0.53	0.17	0.18	0.15	0.61
	28°11’59.45"N	34°22’1.67"E	0.85	0.01	0.01	0.14	0.42
	28°11’57.74"N	34°21’52.10"E	0.63	0.06	0.05	0.13	0.37
	28°11’58.63"N	34°21’55.93"E	0.63	0.04	0.08	0.13	0.41
	28°11’53.92"N	34°21’59.22"E	0.61	0.04	0.03	0.13	0.22
	28°12’43.19"N	34°21’19.42"E	7.10	0.01	0.01	0.23	0.61
	28°12’41.82"N	34°21’21.36"E	5.11	0.17	0.01	0.13	0.18
Old copper mine Wadi Ghorabi-El Hatemeia	28°12’45.98"N	34°21’16.31"E	3.82	0.61	0.16	0.12	0.29
	28°12’43.99"N	34°21’7.05"E	4.16	0.13	0.36	0.16	0.43
	28°12’48.66"N	34°21’13.77"E	4.36	0.26	0.03	0.15	0.65
	28°12’42.14"N	34°21’10.30"E	2.88	0.67	0.09	0.14	0.29
	28°12’39.59"N	34°21’15.20"E	3.46	0.17	0.02	0.13	0.37
	28°12’39.77"N	34°21’20.58"E	2.18	0.29	0.06	0.14	0.60
	28°12’38.42"N	34°21’23.37"E	2.76	0.18	0.02	0.14	0.29
	28°12’39.39"N	34°21’24.92"E	2.78	0.01	0.18	0.12	0.61
	28°12’43.24"N	34°21’29.03"E	2.32	0.24	0.03	0.13	0.40
	28°12’42.32"N	34°21’23.69"E	1.95	0.14	0.15	0.13	0.38
	28°12’47.35"N	34°21’24.25"E	1.76	0.13	0.01	0.13	0.60
	28°12’34.90"N	34°20’43.99"E	1.56	0.18	0.01	0.15	0.52
	28°12’26.90"N	34°20’48.55"E	1.55	0.05	0.01	0.13	0.60
	28°12’36.50"N	34°20’34.37"E	1.49	0.15	0.01	0.13	0.32
	28°12’55.71"N	34°20’25.76"E	1.36	0.06	0.07	0.13	0.23
	28°12’59.05"N	34°20’46.54"E	0.91	0.01	0.07	0.13	0.60
	28°12’49.05"N	34°21’46.46"E	0.93	0.04	0.01	0.21	0.52
	28°12’46.51"N	34°21’56.95"E	0.90	0.05	0.01	0.15	0.60
	28°12’40.82"N	34°21’52.12"E	0.88	0.03	0.03	0.13	0.45
	28°12’34.70"N	34°21’48.53"E	0.86	0.03	0.05	0.17	0.41
	28°12’30.78"N	34°21’40.68"E	0.73	0.19	0.02	0.13	0.42
	28°12’24.08"N	34°21’30.28"E	0.86	0.06	0.02	0.13	0.38
	28°12’25.96"N	34°21’22.44"E	0.93	0.01	0.01	0.13	0.61
	28°12’29.95"N	34°21’20.13"E	0.77	0.11	0.02	0.15	0.33
	28°13’29.00"N	34°19’22.72"E	1.49	0.26	0.09	0.13	0.37
	28°13’34.19"N	34°19’36.73"E	1.72	0.01	0.01	0.13	0.38

	Latitude	Longitude	Cu %	Pb %	Zn %	Au ppm	Ag ppm
Wadi Samra disseminated sulfide area	28°13’23.00"N	34°19’12.07"E	1.22	0.01	0.01	0.13	0.52
	28°13’38.50"N	34°18’58.02"E	0.98	0.06	0.07	0.14	0.29
	28°13’47.45"N	34°19’3.19"E	1.09	0.02	0.01	0.17	0.47
	28°13’46.65"N	34°19’12.40"E	0.87	0.08	0.01	0.19	0.60
	28°13’48.21"N	34°19’23.35"E	0.91	0.01	0.06	0.15	0.42
	28°13’42.08"N	34°19’36.24"E	0.91	0.04	0.01	0.16	0.22
	28°13’35.76"N	34°19’39.61"E	0.85	0.08	0.01	0.19	0.41
	28°13’31.00"N	34°19’32.13"E	0.91	0.01	0.01	0.18	0.38
	28°13’20.26"N	34°19’30.08"E	0.91	0.02	0.01	0.13	0.45
	28°13’15.66"N	34°19’23.43"E	0.91	0.03	0.01	0.12	0.52
	28°13’23.77"N	34°19’49.74"E	0.77	0.05	0.01	0.13	0.29
	28°13’32.32"N	34°19’50.14"E	0.77	0.11	0.01	0.13	0.61
	28°13’23.44"N	34°19’51.82"E	0.87	0.01	0.01	0.13	0.43
	28°13’22.00"N	34°20’0.77"E	0.71	0.11	0.01	0.13	0.36
	28°13’34.53"N	34°20’0.83"E	0.74	0.10	0.01	0.13	0.61
	28°13’24.38"N	34°20’4.90"E	0.68	0.09	0.09	0.15	0.52
	28°13’30.63"N	34°20’15.43"E	0.71	0.06	0.07	1.2	0.03
	28°13’43.55"N	34°20’12.60"E	0.72	0.08	0.01	0.6	0.06
	28°13’59.87"N	34°20’8.48"E	0.59	0.15	0.03	0.16	0.02
	28°13’56.51"N	34°20’2.86"E	0.69	0.03	0.01	1.1	0.09
	28°13’59.51"N	34°19’53.26"E	0.67	0.09	0.03	1.1	0.21
	28°13’57.23"N	34°19’39.81"E	0.63	0.01	0.02	0.48	0.2
	28°14’3.47"N	34°19’23.07"E	0.62	0.01	0.01	0.19	0.2
	28°14’9.10"N	34°19’13.97"E	0.72	0.05	0.05	0.19	0.8
	28°14’2.21"N	34°18’59.66"E	0.64	0.10	0.03	0.24	0.2
	28°13’55.09"N	34°18’52.07"E	0.49	0.16	0.09	0.13	0.13
	28°13’36.42"N	34°18’56.62"E	0.63	0.06	0.05	0.90	0.2
	28°13’6.72"N	34°18’57.74"E	0.53	0.19	0.02	0.60	0.6
	28°13’50.23"N	34°20’19.94"E	0.50	0.23	0.01	0.20	0.6
	28°13’37.03"N	34°20’35.02"E	0.50	0.23	0.01	0.39	0.24
	28°13’40.67"N	34°20’37.00"E	0.57	0.11	0.01	0.50	0.8
	28°13’44.86"N	34°20’40.46"E	0.76	0.01	0.01	0.10	0.18
	28°13’51.54"N	34°20’40.25"E	0.61	0.04	0.03	0.20	0.16
	28°13’45.69"N	34°20’51.03"E	0.55	0.03	0.04	0.50	0.8
Wadi Samra *Stockwork area*	28°14’33.00"N	34°19’37.94"E	5.31	1.32	2.3	0.50	0.829
	28°14’35.09"N	34°19’33.45"E	3.9	1.5	1.19	0.20	0.359
	28°14’31.20"N	34°19’38.97"E	3.15	0.58	1.19	0.40	0.27
	28°14’30.75"N	34°19’23.11"E	2.61	0.55	1.04	0.10	0.13
	28°14’34.75"N	34°19’18.84"E	2.61	0.53	1.04	0.35	0.934
	28°14’39.51"N	34°19’23.76"E	1.84	0.39	1.04	0.30	0.48
	28°14’37.03"N	34°19’29.36"E	1.84	0.31	1.04	0.40	0.29
	28°14’34.27"N	34°19’29.41"E	1.72	0.28	0.59	0.30	0.86
	28°14’31.87"N	34°19’29.91"E	1.4	0.21	0.59	0.21	0.14
	28°14’28.15"N	34°19’28.50"E	1.23	0.18	0.38	0.54	0.219
	28°14’30.24"N	34°19’34.45"E	1.17	0.18	0.38	0.19	0.11
	28°14’34.29"N	34°19’33.10"E	1.07	0.18	0.34	0.51	0.69
	28°14’35.67"N	34°19’34.59"E	1.06	0.16	0.30	0.32	0.12
	28°14’37.06"N	34°19’36.33"E	1.06	0.15	0.24	0.18	0.12
	28°14’34.74"N	34°19’37.53"E	1.01	0.12	0.22	0.15	0.16
	28°14’33.22"N	34°19’37.77"E	0.9	0.09	0.22	0.10	0.18
	28°14’31.48"N	34°19’38.21"E	0.76	0.09	0.17	0.10	0.13
	28°14’32.88"N	34°19’41.05"E	0.72	0.6	0.17	0.19	0.14
	28°14’29.84"N	34°19’44.97"E	0.7	0.03	0.17	0.35	0.171
	28°14’34.56"N	34°19’51.34"E	0.69	0.03	0.17	0.50	0.829
	28°14’37.49"N	34°19’56.20"E	0.63	0.20	0.14	0.20	0.359
	28°14’40.36"N	34°19’56.58"E	0.58	0.02	0.14	0.10	0.17
	28°14’42.57"N	34°19’59.29"E	0.51	0.01	0.14	0.94	0.12
	28°14’45.71"N	34°20’3.14"E	0.50	0.01	0.14	0.35	0.934
	28°14’46.52"N	34°20’4.59"E	0.47	0.10	0.13	0.30	0.28
	28°14’49.99"N	34°20’11.16"E	0.31	0.01	0.13	0.15	0.36
	28°14’47.43"N	34°20’11.64"E	0.26	1.78	0.13	0.40	0.69
	28°14’53.20"N	34°20’13.76"E	0.19	0.04	0.10	0.21	0.24
	28°14’56.13"N	34°20’17.04"E	0.18	0.62	0.10	0.14	0.11
	28°14’46.66"N	34°20’7.34"E	0.11	0.16	0.07	0.19	0.11
	28°14’40.94"N	34°20’28.06"E	0.09	0.13	0.07	0.12	0.152
	28°14’55.50"N	34°20’37.66"E	0.07	0.17	0.07	0.28	0.12
	28°14’29.71"N	34°20’40.34"E	0.07	0.81	0.04	0.15	0.16
	28°14’29.14"N	34°21’8.06"E	0.07	0.72	0.02	0.23	0.28
	28°14’17.15"N	34°21’20.19"E	0.05	0.72	0.02	0.10	0.13
	28°14’6.70"N	34°21’35.19"E	0.04	0.76	0.98	0.19	0.74

Plotting the intrusive plutonic rocks on the Na_2_O + K_2_O versus SiO₂ diagram^55^ places samples in the granodiorite (older granite) and granite (younger granite) fields (Fig. 9j).

All older granite rock samples are high-K calc-alkaline to shoshonitic series, in contrast to the younger granites, which display calc-alkaline to high-K calc-alkaline characteristics^56^ (Fig. 9k). The Al_2_O_3_-CaO-(Na_2_O + K_2_O) alumina saturation diagram^57^ is used to differentiate between the three types of granitic rocks: peraluminous, metaluminous, and peralkaline. Figure 9 indicates that the older and younger granites examined are primarily peraluminous to weakly metaluminous, situated close to the metaluminous and peraluminous boundary.

According to the Chappell and White^58^ K₂O versus Na₂O diagram, the older and younger granites fall within the I-type granite field (Fig. 8m), which was supported by the SiO_2_ content and mineralogy (occurrence of hornblende and biotite).

Tectonic discrimination diagrams Nb vs. Y and Rb vs. Y + Nb^59,60^ (Figs. 9n and o) show that the older granitoids cluster in the volcanic arc granite (VAG) field, consistent with subduction-related magmatism. In contrast, the younger granites plot within the within-plate granite (WPG) field.

### Mineralization styles

**Fig. 10:**
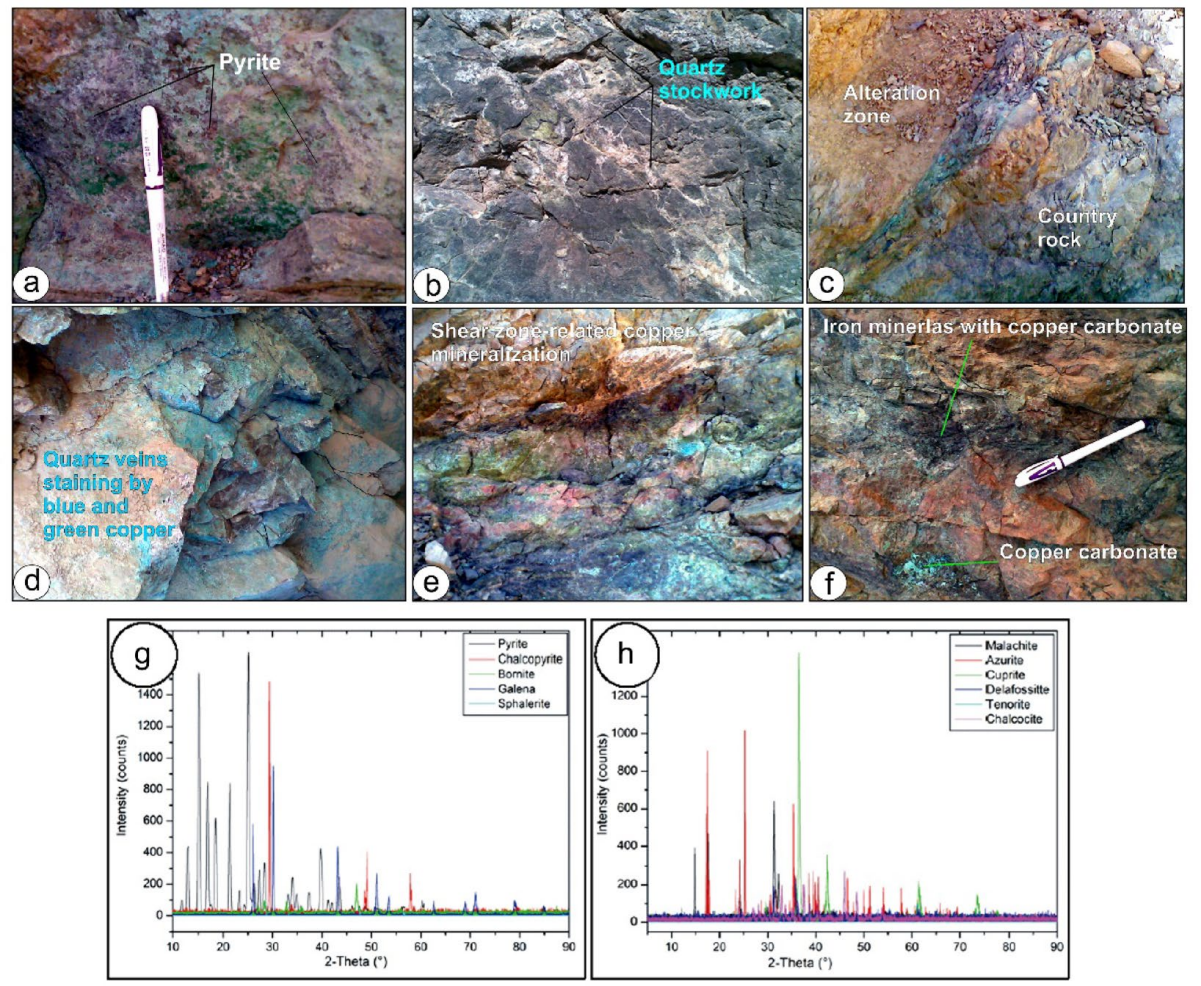
(**a**) Pyrite-bearing metarhyolite-ignimbrite sheets showing disseminated sulfides, (**b**) Stockwork made up of network of intersecting, thin (few centimeters thick), randomly oriented quartz veins, (**c**) Alteration zone at the northern bank of Wadi Ghorabi-El Hatemeia, (**d**) Quartz vein traverse the alteration zone and staining by green and blue copper carbonate, (**e**) Copper ore hosted mainly along fractures system related to shear zone traverse graywack and pyroclastic rocks at Wadi Khashm El-Fakha, (**f**) Gossan bodies mineralization along Wadi Tarr mineralization where copper carbonate form thin bands mixed with iron minerals and green staining. X-ray diffractograms of the studied ore mineralizations showing (g) a variety of primary minerals mainly pyrite, chalcopyrite, bornite, galena and sphalerite, (**h**) secondary minerals mainly malachite, azurite, chalcocite, cuprite, delafossitte, and tenorite.

Disseminated pyrite mineralization occurs within the metarhyolite–ignimbrite units, with a sulfide patch pattern (Fig. 10a). Petrographic and field observations indicate that sulfide minerals (pyrite, chalcopyrite, bornite) constitute 20–40 vol% of the mineralized zones in metarhyolite–ignimbrite units, as estimated by point-counting in thin sections.

In Wadi Samra, sulfide mineralization is mainly represented by quartz stockwork, whereas in Wadi Khashm El-Fakh it appears less abundantly. It is found as a network of intersecting, thin (a few centimeters thick), randomly oriented quartz veins that make up stockwork (Fig. 10b), which spans approximately 0.15 km^2^. The stockwork quartz veins show a variety of trends, encompassing NNW-SSW, E-W, and NW-SE orientations. In Wadi Samra, quartz stockwork cuts cross metasediments but rarely cuts cross metavolcanics and pyroclastic rocks; on the other hand, in Wadi Khashm El-Fakha, the quartz stockwork cuts cross pyroclastic rocks of the metavolcanics. Generally, disseminated sulfides dominate the stockwork area, primarily as chalcopyrite and pyrite, with occasional 1–3 cm occurrences of galena and sphalerite. Iron oxides and malachite stain quartz veins and veinlets of the stockwork area; these secondary minerals are alteration products of the primary minerals. Other alteration minerals include covellite and chalcocite.

The historic copper workings in the upper Wadi Ghorabi-El Hatemeia are associated with two prominent hydrothermal alteration zones, reflecting structurally controlled fluid flow. The larger zone, developed along the northern bank, strikes N45°E and is aligned with NNW–SSE-trending fractures, which acted as primary conduits for mineralizing fluids.

The alteration zones, notable for their vivid staining from malachite (green) and azurite (blue) copper-carbonate minerals, are traversed by a 2 m-thick quartz vein. These zones extend for 500 m along strike (Figs. 10c and d) with a highly variable thickness of 2.5 to 18.5 m^28^. The quartz vein extends about 400 m in length then disappears at the foot of the wall rock, extending in the same direction of the alteration zone. On the opposite side, a second alteration zone is exposed, characterized by malachite and azurite mineralization with a complete absence of quartz veins. Here, mineralization is structurally controlled, occurring as fracture-filling mineralizations along the intersections of NNW–SSE and NE–SW fracture systems within the volcanic-sedimentary host rocks.

The copper mineralization is dominated by structural fractures rather than lithological or stratigraphic ones. This copper ore, hosted mainly along a fracture system, may be related to a shear zone that traverses graywack and pyroclastic rocks that crop out along Wadi Khashm El-Fakha, between Wadi Tarr and Wadi Ghorabi-El Hatemeia (Fig. 10e). Three alteration zones, corresponding to the NW-SE trend of the mineralized shear zone, host the alteration minerals kaolinite, carbonate, chlorite, and sericite. Copper mineralization occurs as copper carbonate (mainly bright green to blue) malachite, azurite, crysocolla, and cuprite mixed with covellite and chalcocite. These secondary minerals show relics of primary sulfide minerals of chalcopyrite, galena, and sphalerite.

Along Wadi Tarr, mineralization is expressed as elongated gossanous bodies striking NE–SW, with dimensions reaching hundreds of meters in length and tens of meters in width. The linear shape of the gossan bodies and their parallelism in the same direction as the main foliations of the host rocks indicate the structure-controlled effects in their formation. These gossan bodies are distinguished by their varying iron content, from massive iron minerals to those with only superficial iron staining. Copper mineral forms thin bands mixed with iron minerals and green staining (Fig. 9f). It is common to observe the silicification of volcanic rocks, particularly the pyroclastic varieties. The ore minerals are represented by a mixture of primary and secondary sulfides, mainly chalcocite, covellite, bornite, malachite, and azurite.

## Discussion

### Lithological and structural mapping

The application of selective colored imagery from Landsat-8 demonstrated its utility in discriminating rock units and improved structural mapping in the Samra area by highlighting sharp contacts within the volcanic sequences (Fig. 3). (Fahmy et al.[53]^55^ utilized Landsat-8 to map and distinguish the volcanic sequences east of the study area, employing various color composites such as FCC 761 and BR 2/1, 3/4, 4/7, and PC 123 and 136 in RGB mode. In the present study, the selected transformed images, including FCC 567, BR 1/3, 1/7, 3/6, MNF 234, and PC 213 (Fig. 3), effectively delineated the volcanic units and differentiated them from the surrounding country rocks, such as conglomerates and granitoids. The entire range of colored imagery showcased tonal variations, particularly in FCC 567 and BR 1/3, 1/7, 3/6-RGB, highlighting specific rock types like rhyodacite and porphyritic dacite, which are associated with copper, as well as albitite. These tonal differences helped separate these units from others in the study area. Additionally, valleys, thrust contacts, and fault displacements were monitored through the aforementioned colored composites (Fig. 3a and b). The identified rock units and their spatial distribution were validated using field and petrographic data, which contributed to the development of a highly accurate geological map of the specified area (Fig. 1b).

### Mineralogy

Mineralogical investigations, field observations, and X-ray diffraction (XRD) analyses reveal that copper mineralization in the Wadi Samra area occurs as primary sulfides and secondary oxidation products, primarily hosted in metarhyolite–ignimbrite units. The dominant primary sulfides are pyrite, chalcopyrite, and bornite, with secondary minerals including malachite, azurite, and covellite (Figs. 10g and h).

**Fig. 11:**
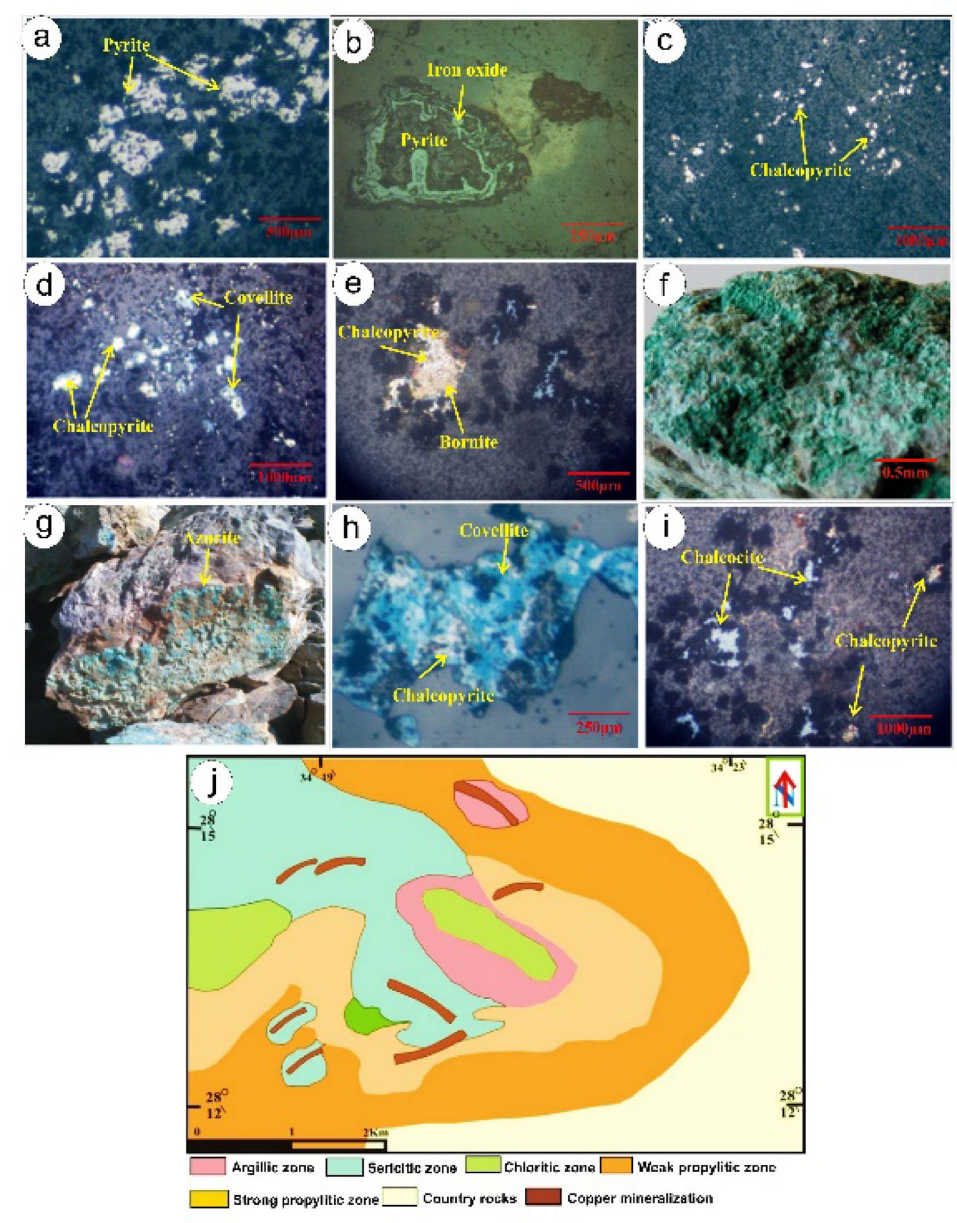
(**a**) a random distribution pyrite cube in pyrite-bearing rhyolite- ignimbrite sheets; (**b**) Pyrite disseminated in quartz veins and altered to limonite and/or hematite, Old Copper mined area; (**c**) Chalcopyrite occurs mainly either as subhedral to anhedral fine-grained crystals in the quartz vein; (**d**) Chalcopyrite altered along grain boundaries to and covellite, (**e**) Subhedral bornite crystals intergrown with chalcopyrite; (**f**) Botryoidal Malachite aggregates; (**g**) Massive azurite in volcanic and sedimentary rocks; (**h**) Covellite occurs replacement of chalcopyrite mineral, note relics of chalcopyrite can still be observed; (**i**) ) Chalcocite occurs as thin stringers or cracks falling, (**j**) Alteration map of Samra area showing hydrothermal alteration zoning pattern from inner serictic to outer propylitic zones.

Primary sulfides Pyrite is the most abundant sulfide, comprising 20–40 vol% of mineralized zones in metarhyolite–ignimbrite, as estimated by petrographic point-counting (Figs. 11a and b). It occurs as euhedral to subhedral cubic crystals, often intergrown with subordinate chalcopyrite and bornite. This high volumetric sulfide content contrasts with lower base metal concentrations (7.1 wt% Cu) due to the prevalence of pyrite, a low-Cu sulfide mineral.

Chalcopyrite forms small, irregular crystals within quartz veins and stockworks, occasionally showing myrmekitic intergrowths with bornite (Figs. 10c and d). Bornite appears as subhedral to anhedral crystals, commonly associated with chalcopyrite, and locally forms veinlets or exsolution textures (Fig. 11e).

Secondary minerals Supergene alteration has produced malachite, the most common copper carbonate, forming botryoidal aggregates and fracture fillings in volcanic rocks (Fig. 11f). Azurite, less abundant, occurs as fine-grained aggregates in cavities (Fig. 11g). Covellite, a secondary sulfide, forms indigo-blue rims around chalcopyrite and bornite due to supergene enrichment (Fig. 11h). Minor chalcocite appears as thin veinlets or coatings around primary sulfides (Fig. 11i).

Accessory and Alteration Minerals: Magnetite as accessory phase in quartz-diorite, indicating oxidized conditions. Minor minerals (e.g., galena, sphalerite, delafossite, tenorite and and copper iron oxide Fe_2_CuO_4_) were detected by XRD but are less significant to the mineralization.The mineral assemblage reflects three processes: (1) primary hydrothermal/magmatic sulfide formation (pyrite, chalcopyrite, bornite); (2) supergene oxidation forming copper carbonates and oxides (malachite, azurite); and (3) secondary sulfide development (covellite, chalcocite) due to near-surface leaching.

### Hydrothermal alteration zone

The Wadi Samra area is characterized by extensive hydrothermal alteration processes, primarily affecting volcano-sedimentary rocks, which have led to the formation of various alteration zones (Fig. 11j).

Ore zones, particularly copper and gold mineralizations, are closely linked to the hydrothermal alterations, where it is found that the hydrothermal alteration extends several meters around the ore mineralizations. Several hydrothermal alterations are observed along the NE-SW fault/shear zone. Field and petrographic studies have identified three alteration zones with gradually altering boundaries; they are sericitic, argillic, and propylitic alteration zones.

#### Sericitic (potassic) alteration

**Fig. 12:**
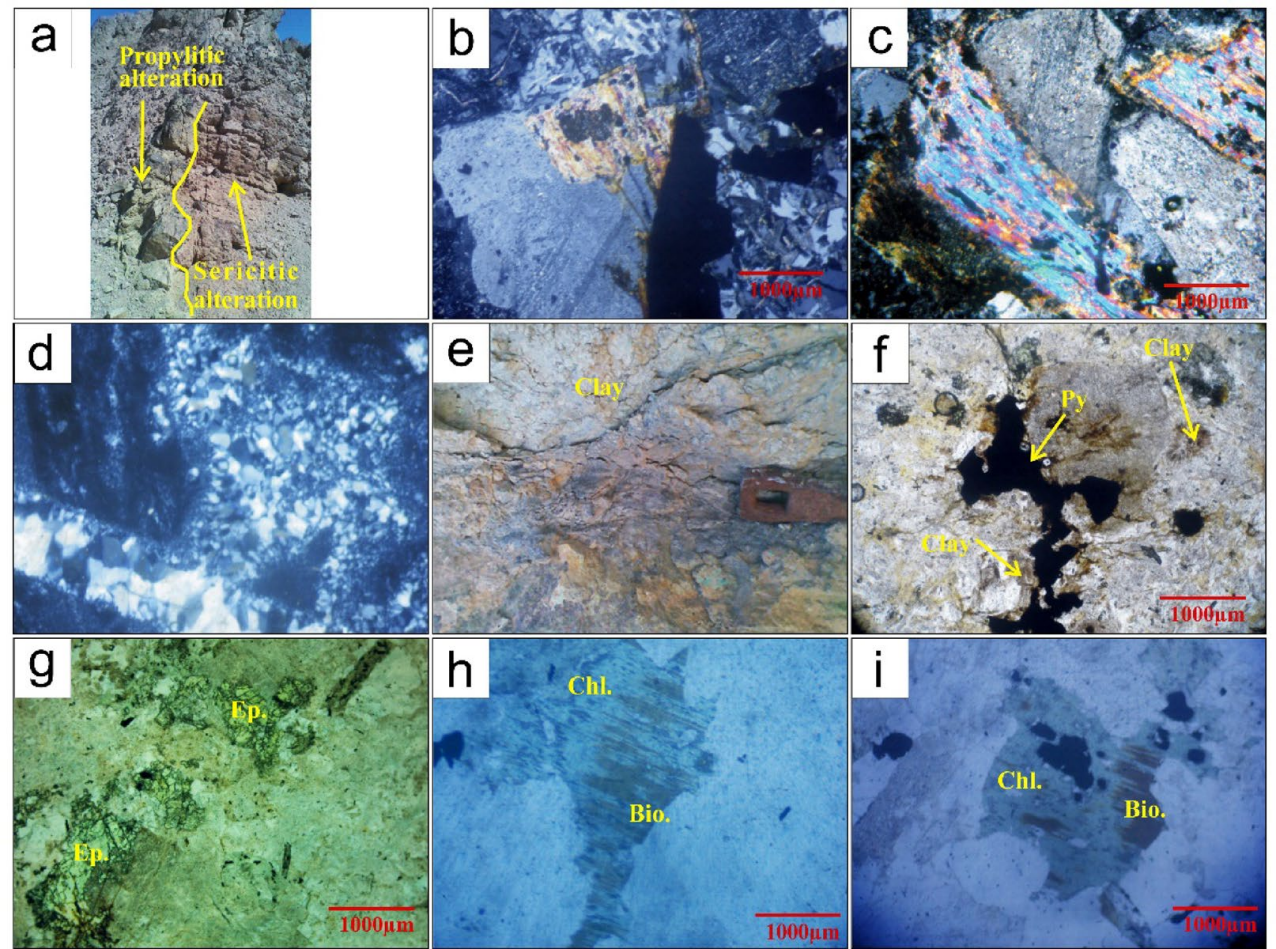
(**a**) Pink potassic and greenish color propylitic alteration zones; (**b**) Secondary biotite replaces hornblende, (**c**) Secondary muscovite (muscovitization) is widespread alteration replaces both K-feldspars and Plagioclase, (**d**) Secondary quartz made up of fine-grained anhedral interlocking grains, develops few millimetres nests and veinlets, (**e**) Grayishwhite argillic alteration, (**f**) Clay minerals and associated with oxidized pyrite in argillic alteration, (**g**) Epidote replaces plagioclase in propylitic alteration zone, (**h**) Chlorite place ferromagnesian minerals particularly hornblende and biotite, (**i**) Chlorite formed due to replacement of biotite mica in chlorite alteration zone. Py; Pyrite, Chl; Chlorite; Bio; Biotite, Ep; Epidote.

In the Samra area, the granodiorite units are extensively overprinted by sericitic alteration, typical of phyllic zones in porphyry systems. Sericite, secondary K-feldspar, muscovite, fine-grained quartz, and disseminated pyrite characterize this alteration assemblage. In the granitic rocks, plagioclase undergoes partial alteration to secondary K-feldspar, forming a potassium-enriched pink alteration zone confined to areas surrounding quartz veins (Fig. 12a). Secondary biotite has replaced the hornblende. It can take the form of fine-grained linear biotite or dissemination forms (Fig. 12b). Secondary muscovite (muscovitization) is a widespread alteration of both K-feldspars and Plagioclase, where they are replaced by coarse-grained flaky muscovite (Fig. 12c). The secondary muscovite has replaced the feldspar minerals with no predominant orientation along the feldspar crystal cleavage. Also, the secondary muscovite in flaky grains is formed through the progressive replacement of earlier sericitized minerals. Furthermore, secondary quartz, which is made up of small, interlocking anhedral crystals, develops nests that are a few millimetres in size (and/or stockwork, veinlets (Fig. 12d). Pyrite represents the dominant authigenic phase within the alteration zone, occurring as disseminated grains that display variations in both size and crystal habit.

#### Argillaceous alteration

In the field, argillaceous alteration is identified by a grayish white color (Fig. 12e). At the same time, locally, it has light yellow to light brown colors due to the abundance of iron oxide-hydroxides. Argillaceous alteration affects the metavolcanic–sedimentary country rocks and the intrusive granitic units. This alteration zone is characterized by the development of quartz, kaolinite, montmorillonite, chlorite, and calcite, accompanied by minor amounts of illite and hydrated iron oxides. The potash feldspars of the outer limit of the sericitic alteration zone are slightly to intensely altered to this clay mineral assemblage. Where feldspars are first altered later into sericite minerals, then the sericite is transformed into clay minerals. In specific samples, the transformation of sericite into clay minerals such as kaolinite and illite appears incomplete. This is evidenced by the notable coexistence of residual sericite alongside newly formed clay minerals. Because the clay mineral content in the other areas is locally quite significant, it is designated as a distinct argillaceous zone on the alteration map, where oxidized pyrite (Fig. 12f) and iron oxide-hydroxides are prevalent; copper minerals are scarce in this alteration zone.

#### Propylitic alteration

This alteration zone occupies mostly the outermost alteration zones around mineralization and fades out gradually into the fresh rocks. Propylitic alteration predominantly affects the volcaniclastic and intermediate volcanic units encircling the granitic porphyry, marking the outermost halo of the hydrothermal system. This zone is clearly distinguished from the inner argillic and phyllic alteration domains.

Weakly propylitic alteration is characterized by epidote, chlorite, albite, fine-grained calcite, and magnetite. In this process, plagioclase is altered to epidote and calcite (Fig. 12g), while epidote and chlorite selectively replace ferromagnesian minerals, particularly hornblende and biotite (Fig. 12h). Magnetite is widely distributed in this alteration zone and occurs as disseminated grains within the altered rocks.

Strongly propylitic alteration was represented by a soft, bright green zone (Fig. 12a). It occurs close to the sericitic alteration zone, where the chloritic zone locally overlaps them. This advanced propylitization zone is made up mainly of epidote and chlorite.

#### Chloritic alteration

Chlorite overprinted hydrothermal alteration is formed upon the strongly propylitic alteration. Mineral assemblages of chlorite-epidote and quartz-sericite characterize this type of alteration. Chlorite forms thin zones along the propylitic alteration contacts, layered aggregates within propylitic alteration, or as flake grains distributed within the propylitic zone (Fig. 11j). Chlorite alteration is formed due to the replacement of biotite mica (Fig. 12i).

### Geochemistry of the alteration zone

**Fig. 13:**
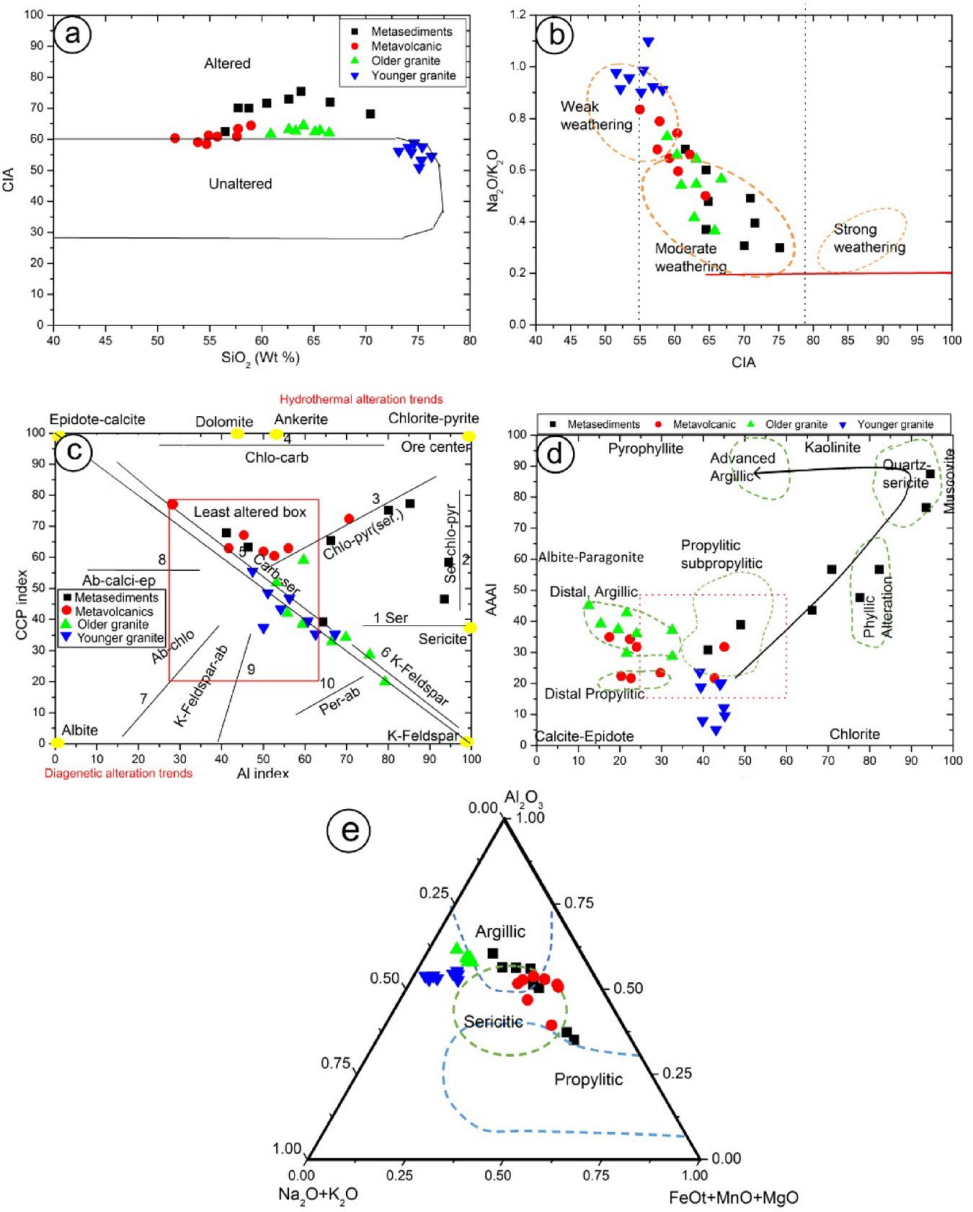
(**a**) Alteration filter SiO_2_ vs. CIA diagram^61,62^; (**b**) CIA vs. Na_2_O/K_2_O diagram^63^, (**c**) The alteration box AI vs. CCPI^64^, (**d**) alteration index (AI) versus advanced argillic alteration index (AAAI)diagram (Williams and Davidson, 2004).

To distinguish altered samples from reasonably fresh samples from the Samra district, this study employed the alteration filter SiO2vs. CIA diagram^61,62^. This diagram shows that all the metasediments, older granite, and most metavolcanic rocks are altered. In contrast, the younger granite samples are plotted inside the unaltered field^63^ (Fig. 13a) applied the CIA vs. Na_2_O/K_2_O diagram to identify the chemical weathering intensities of the altered rock samples, plotting samples in this diagram show that metasediments, older granite, and a part of the metavolcanics suffered from moderate alteration. The younger granite and the remaining metavolcanic samples were weakly altered (Fig. 13b).

The alteration box AI vs. CCPI diagram^64^ is a helpful tool for differentiating diagenetic overprints from the hydrothermal signature. The CCPI vs. Al diagram depicting the alteration zone samples (Fig. 13c) shows that most samples are located in the hydrothermal alteration side. Granitic samples display K-feldspar and carbonate-sericite trends. Metasediments and metavolcanic display the chlorite-pyrite-sericite, while the sericite trend is followed by the metasediments only.

Williams and Davidson^65^ used the alteration index (AI) vs. the advanced argillaceous alteration index (AAAI) to assess the intensity of geochemical alteration related to the ore-forming fluids. The older granite and the metavolcanic samples are plotted in the distal argillic and distal propylitic alteration zone, the metasediments show gradually increasing of AI value toward zone of phyllic alteration, in addition, number of samples display Al values > 90 reflects lower temperature alteration minerals such as muscovite and kaolinite in the quartz- sericite alteration zone (Fig. 13d). The AKF ternary diagram^66^ was used to determine type of alteration, where the altered samples from the different rock units are distributed between argillaceous, sericitic, and propylitic fields (Fig. 13e).

### Copper potential zones from remotely sensed data

Combining different datasets—such as lithological, structural, and remote sensing information—is considered one of the most powerful approaches for identifying areas with high mineralization potential. To designate the high-potential zones for copper and other ore minerals, an integration of optical and SAR data with field data has been performed, demonstrating its high efficiency as evidenced by previous studies conducted by various researchers^13–17,22,67^ which aims to highlight and map these potential zones.

**Fig. 14:**
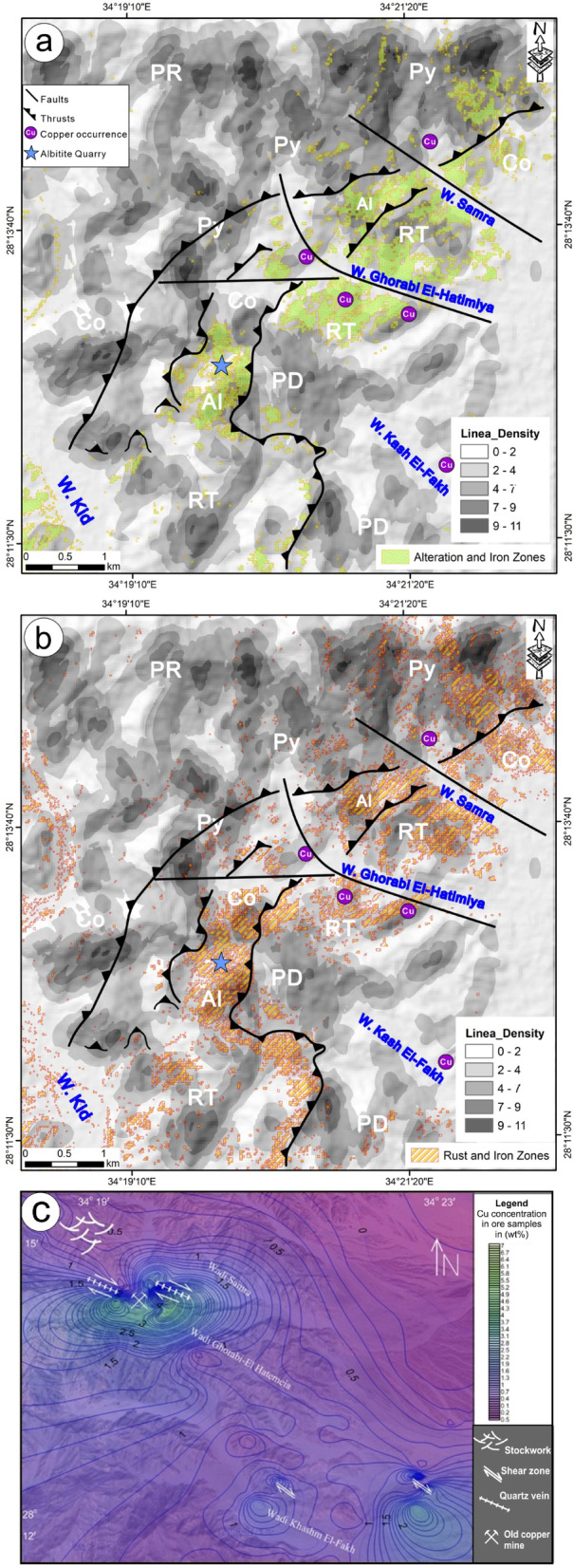
Potential copper maps; (**a**) By Landsat-8 and (**b**) By ASTER. Rock abbreviations see Fig. 3. (**c**) Cu-anomaly map (created by interpolations of Cu (wt%) concentration in 120 ore samples collected from the mineralized area). These figures were created and processed by ENVI v. 5.6.2. software: https://www.l3harrisgeospatial.com/Software-Technology/ENVI), which is mainly utilized for image processing, and ArcGIS Desktop 10.8. (https://www.esri.com/en-us/arcgis/products/arcgis-desktop/overview/).

The distribution of hydrothermal alteration zones, recognized as advantageous regions for ore mineralization, is a crucial factor in exploring ore mineralization. Furthermore, a high density of lineaments, which serve as pathways for mineralized hot fluids, may indicate a higher degree of rock fracturing closely linked to mineralization. Consequently, the lineament density map derived from the S1A radar data was utilized to illustrate the spatial distribution of lithological units, structural features, and alteration zones identified by ASTER and Landsat-8 (Figs. 14a and b). The analysis of the alteration and iron zones detected through the BRs and SAM methods on both Landsat-8 and ASTER (Fig. 4) indicated that they coincide and align with the identified high-density alteration zones (Figs. 4 and 5). The synthesis potential copper maps designated by ASTER and Landsat-8 revealed that the high-density zones coincide with regions of moderate to high lineament density, characterized by rhyodacite and porphyritic dacite composition, which show a high frequency of structural elements. Furthermore, the distribution of copper occurrences alongside the albitite quarry coincided with the recognized alteration zones (Figs. 14a and b). This coincidence illustrates the accuracy of the identified alteration zones, which were substantiated by field, petrographical and geochemical investigations.

The potential maps (Fig. 2a and b), generated from Landsat-8 and ASTER data, illustrate the spatial distribution of Al, CO3-OH, iron, and rust zones. These are key indicators of main alteration zones like argillic, phyllic, propylitic, and gossan zones, where ore mineralizations such as gold, copper, and lead may occur in the Wadi Samra area. Field studies confirmed that these alteration zones extend several meters around the ore deposits and along the NE-SW fault/shear zone, with boundaries gradually changing. They have also been verified through petrological and geochemical analyses of representative altered samples collected from the study area. Furthermore, the maps reveal that the altered zones form a NE-striking belt mainly composed of rhyodacite, surrounded by NE-thrust contacts, which could be high-potential zones for copper exploration.

### Ore zoning and copper distribution

One hundred nineteen samples of mineralization were collected from the mineralized zones in Wadi Samra, and its surrounding areas (Table 4). The samples were analyzed for copper, lead, zinc, gold, and other trace elements. Figure 14c shows the Cu-anomaly map of the analyzed samples. In the study area, copper distribution within the mineralized rock units shows that the stockwork zone is enriched in copper and locally associated with elevated zinc values. Copper concentrations range between 0.03 and 5.31 wt%, averaging 1.05 wt%. Disseminated sulfide’s location displays the Cu values lower than those in the stockwork’s location, ranging from 0.55 to 1.49 with an average of 0.816292 wt%.

Samples from the alteration zones at Wadi Ghorabi-El Hatemeia show copper (Cu) concentrations ranging from 0.77 to 7.1 wt%, averaging 2.24 wt%, and are associated with elevated gold values up to 0.23 ppm. In these later localities, the high volumetric sulfide content (20–40 vol%) is attributed to the abundance of pyrite, which has a low Cu content, while chalcopyrite and bornite contribute to the base metal concentrations (up to 7.1 wt% Cu). This distinction reflects the mineralogical composition of the mineralization, where gangue minerals and pyrite dominate over Cu-rich sulfides. In Wadi Khashm El Fahk, Cu concentrations range from 0.614 to 1.44 wt%, with an average of 0.85 wt%. Copper is the dominant metal at this location, with locally elevated zinc (Zn) values reaching up to 0.182 wt%. Thus, the chemical analyses of 119 ore samples, however, show base metal concentrations (Cu, Zn, Pb) ranging from 0.03 to 7.1 wt%, with an average Cu content of 1.05 wt% (Table 3), reflecting the dominance of pyrite over Cu-rich sulfides.

### Genetic consideration

**Fig. 15:**
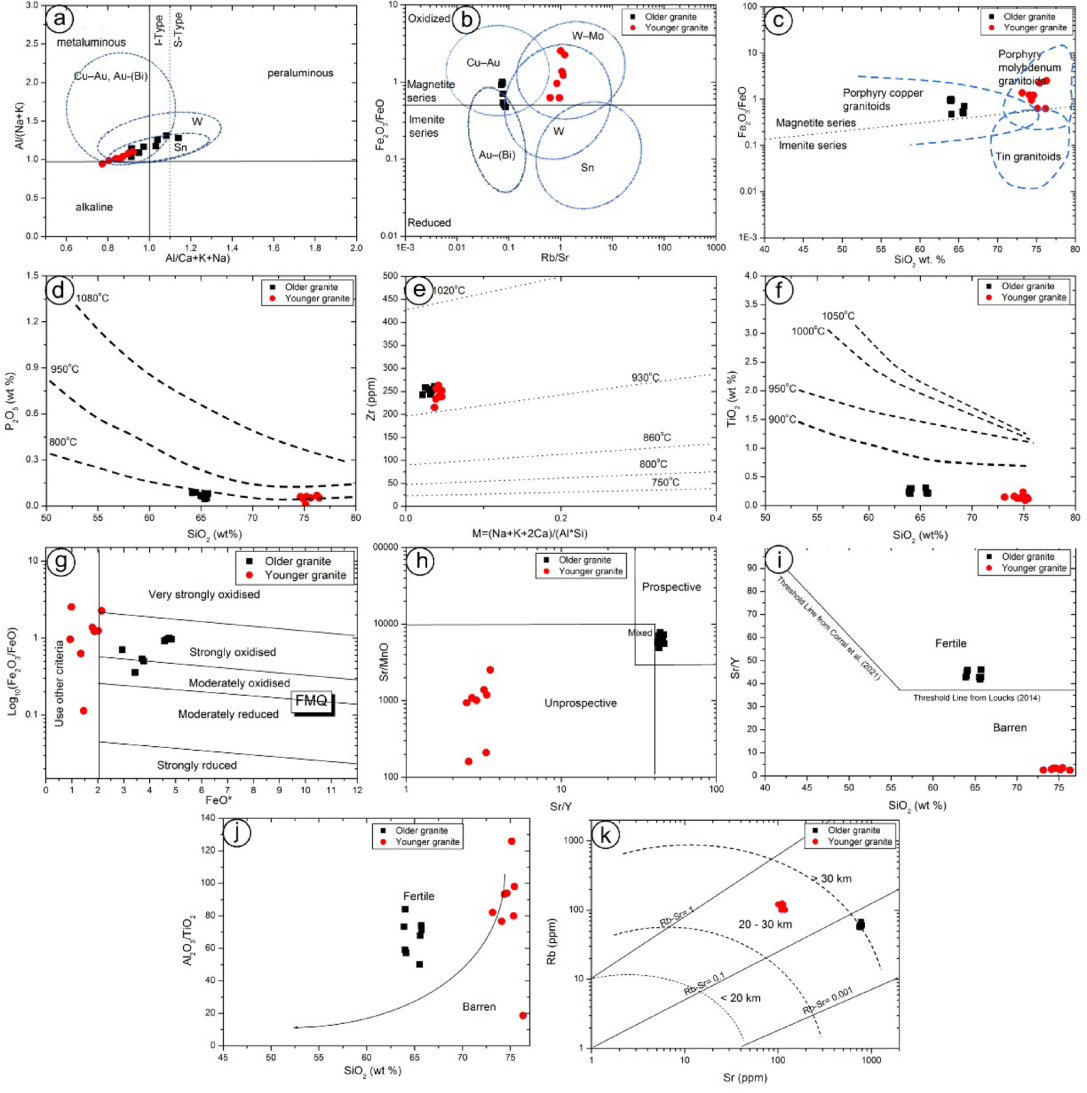
(**a**) A/NK vs. A/CNK bi-plot^71,72^ where granite samples from the study area plot within Cu-Au, Au-(Bi) dominating flied, (**b**) Degree of fractionation (Rb/Sr) vs. the oxidation state (Fe_2_O_3_/FeO)^71,82^. (**C**) Fe_2_O_3_/FeO versus SiO_2_^85^, (**D**) P_2_O_5_ vs. SiO_2_^86^,(**e**) Zr (ppm) vs. M=(Na + K + 2Ca)/(Al*Si) diagram^87^,(**f**) P_2_O_5_ and TiO_2_^87^. (g) Redox indicator expressed as Fe_2_O_3_/FeO vs. FeO*^90^, (**h**) Sr/MnO vs. Sr/Y bi-plot^90^, (**i**) Sr/Y vs. SiO_2_plot^91,92^, (**j**) Al_2_O_3_/TiO_2_ratio versus SiO_2_^91^, (**k**) Rb-Sr diagram^93^.

The South Sinai area hosts notable copper and gold mineralization, with molybdenum occurring in smaller amounts. It belongs to a wider Neoproterozoic metallogenic corridor in southern Sinai, where Cu-Au and base metal sulfide mineralization is genetically associated with high-K calc-alkaline, porphyritic intrusions emplaced within volcanic ring complexes^5^;^10,68–70^. The Neoproterozoic volcano-sedimentary sequence in the study area has been underplated and intruded by multiphase granitoids. These plutons record a magmatic evolution from gabbro-diorite to granodiorite and finally granite^11,69^. Using the plot of the A/NK vs. A/CNK^71,72^ the majority of the granite samples from the study area are plotted within common areas of three ellipses, which characterize the Cu-Au, Au-(Bi) dominating, W, and Sn granite (Fig. 15a). Based on these results, the granitoids from Samra area have a high potential of metallic ores, being especially rich in Cu and minor Au.

In the Samra area, geochemical data indicate that the granitoid intrusions are high-K calc-alkaline to shoshonitic in composition and were emplaced in a volcanic arc setting, consistent with the regional Cu-Au-Mo mineralization signature^73–81^. A plot of Rb/Sr (fractionation index) versus Fe₂O₃/FeO (redox indicator) shows that the Samra granitoids are both highly fractionated and strongly oxidized, positioning them within the magma field typically linked to porphyry Cu systems^71,82^. Their geochemical characteristics closely parallel those of fertile, arc-related intrusions globally (Fig. 15b). One of the most important variables influencing the type of metals precipitating in mineralizations is the redox state of granitic magmas^82–84^. Sinclair^84^ used Fe_2_O_3_/FeO versus SiO_2_ plot diagram to differentiate between the oxidized, magnetite-series and the reduced, ilmenite-series plutons and their influences on the metal contents of related mineralizations. This plot shows that all mineralizations are associated with a magnetite-series and most older granite samples lie within the porphyry copper granitoids field. Younger granite samples lie within the porphyry molybdenum granitoids field (Fig. 15c). Some authors used different methods to estimate the crystallization temperature of the granitoids.

A correlation between magma silica content and apatite saturation was established by Watson and Green^85^, who demonstrated that P_2_O_5_ and SiO_2_ concentrations in granitic rocks can serve as a geothermometer for high-K calc-alkaline magmas. The P_2_O_5_ vs. SiO_2_binary diagram (Fig. 15d) shows an 800 °C isotherm trend for the studied granitic rocks. Because zircon and apatite are highly refractory and often survive partial melting of the crust, their residual presence or the Zr content in granitic melts provides a robust proxy for magmatic temperature. This method can also be applied to the Zr (ppm) vs. M=(Na + K + 2Ca)/(Al*Si) diagram of Watson and Harrison^86^. In Fig. 15e, the estimated temperatures of granitic rocks are slightly above 900 °C. Kwékam et al.^87^ used whole-rock P_2_O_5_ and TiO_2_contents to estimate crystallization temperatures of granite rocks, and temperatures obtained using this diagram indicate values below 900 °C (Fig. 15f). The obtained temperatures are close to the temperatures estimate by Lebda et al.^88^; 850 °C) using Log O2–temperature estimation proposed by Wones and Eugster^89^ of the biotite from Shahera granites located to the north of the study area.

Moderate to high oxidation is a key fertility criterion for magmas hosting porphyry Cu- with minor Au systems. As demonstrated by, oxidized conditions promote the conversion of sulfide (S²⁻) to sulfate (SO_4_^2-^) during partial melting, enhancing the solubility and mobilization of chalcophile elements such as Cu, Au, and Mo into the melt^90–92^. The Fe₂O₃/FeO ratio serves as a reliable empirical proxy for magma redox state. In the studied granodiorite porphyries, these ratios indicate that the parental magmas experienced moderate to strong oxidation (Fig. 15g). These high Fe_2_O_3_/FeO ratios observed in the Samra granitoids are consistent with magnetite-series granitoids (Fig. 15b, c), which are typically associated with porphyry Cu- with minor Au mineralization under oxidized conditions.

The generation of porphyry Cu ± Au mineralizations is fundamentally linked to the fertility of the parent magma, with a high abundance of chalcophile elements being a prerequisite^93^. Fertile arc magmas are characterized by specific geochemical signatures: low loss on ignition (LOI < 3.5%), high Sr/Y (> 35), elevated V/Sc ratios (> 32.5), and intermediate to felsic SiO_2_ content (58–70 wt%)^94^. The older granitoids in the Samra area meet al.l these criteria—LOI < 1.8, Sr/Y > 41.8, and SiO_2_ = 63.9–65.78 wt%—indicating a high degree of magmatic differentiation and oxidation, consistent with a convergent-margin setting conducive to porphyry copper formation. Recent studies used a bi-plot diagram to verify the fertile granitic intrusions of the porphyry copper mineralization.

The usefulness of the Sr/MnO vs. Sr/Y biplot for differentiating between the productive and unproductive porphyry copper systems was documented by Ahmed et al.^95^. Plotting the older and younger granite samples for Samra area show that the older granite is prospective while the younger one is unprospective (Fig. 15h). Sr/Y vs. SiO_2_ plot^94,96^ indicates that the older granite samples are fertile while the younger granite samples are barren (Fig. 15i). Al_2_O_3_ /TiO_2_ratio versus SiO_2_ diagram^94^ displays the composition of the older granitoids plotting in fertile field and the younger granites composition plotting in barren field (Fig. 15j). According to Rb-Sr diagram^97^, the granitic rocks under study mainly were formed from magma that originated at a depth of 20–30 km (Fig. 15k).

The copper mineralization in the Samra area reflects a porphyry Cu- with minor Au system that is structurally and geochemically linked to skarn-type mineralizations, forming part of an integrated magmatic-hydrothermal complex. This magmatic-hydrothermal architecture, where porphyry and skarn mineralization coexist, is comparable to Late Paleozoic systems in the Central Asian Orogenic Belt^98^, suggesting similar tectonic controls and fertile, oxidized arc magmatism in Neoproterozoic southern Sinai.

## Conclusion

- The Wadi Samra region, which is part of the Kid metamorphic belt and the Tarr Complex, exhibits a variety of lithologies such as low-grade lavas, ignimbrites, volcanic breccias, tuffs, mudstones, and schists.
- Lava flows are predominantly represented by porphyritic rhyolites, rhyodacites, dacites, andesites, and less frequently occurring andesitic basalts. These rocks are intruded by multi-phase granitoids that range from gabbro-diorite to quartz-diorite, granodiorite, syenogranite, and alkali granite. Notably, the granitoids, the quartz-diorite and granodiorite phases, are linked to porphyry copper mineralization.
- This study employs Landsat-8 spectral bands and ASTER data to delineate the distribution of ferrous and ferric iron oxides within copper belts. The spectral characteristics of hematite, jarosite, biotite, muscovite, chlorite, and epidote were identified using the SAM technique. The surface distributions of rust zone minerals were classified using the USGS spectral library and a rule threshold of 0.650. The rust zones were integrated using ArcMap software. A geometrically reassessed Sentinel-1 A image and an adaptive enhanced Lee filter were utilized to improve the visibility of structural lineaments. The principal components of PC1, PC2, and PC3 images were analyzed to identify significant structural and linear features. The azimuth frequency diagram indicated that the predominant trend of lineaments is NE, followed by ENE, NNE, and NW trends. The study area is marked by moderate concentrations of lineaments throughout the volcanic sequence and syenogranite rocks.
- The Tarr Complex, situated in the Wadi Samra area, is defined by three distinct phases of deformation: D1, D2, and D3. The interactions among rhyodacitic tuffs, pyroclastics, albitite, and porphyritic dacite are affected by thrust faults that dip towards the northwest. Although folds are infrequent in the metavolcanics of the Tarr Complex, sparse asymmetric folds are primarily found within shear zones. The northern part of the Wadi Samra region contains a northwest-dipping thrust zone, likely indicative of geological activity.
- Copper mineralization is represented by quartz veins, stockwork zones, disseminated sulfides in metarhyolite sheets, alteration zones associated with quartz veins, shear zone-related alteration, and gossan bodies. Primary copper mineralization includes sulfides such as pyrite, chalcopyrite, and bornite, with minor galena and sphalerite. Secondary minerals include malachite, azurite, covellite, chalcocite, delafossite, tenorite, and iron oxides. The porphyry copper mineralization is associated with hydrothermal alteration zones, including sericitic, argillic, propylitic, and chloritic types. These zones affect both the volcanic and sedimentary rocks and the intruded granites.
- The mineralized granitoids, primarily quartz-diorite and granodiorite, are high-K calc-alkaline to shoshonitic, peraluminous to weakly metaluminous, and belong to the I-type granites developed in a volcanic arc setting. These granitic rocks crystallized from magma at temperatures between 800 °C and 900 °C and depths of 20–30 km.
- The synthesis potential copper maps indicated that the high-density zones coincided with moderate to high lineament density regions, characterized by rhyodacite and porphyritic dacite composition. The distribution of copper occurrences alongside the albitite quarry coincided with the recognized alteration zones, demonstrating the accuracy of the identified alteration zones.
- Geochemical analyses indicate high potential for metallic ores rich in copper and gold. The granitoids are fertile and contain significant chalcophile elements. Geochemical plotting shows similarities between these granitoids and igneous complexes associated with porphyry Cu- with minor Au mineralization. The granitic rocks met the criteria and favorable conditions of productive porphyry Cu- with minor Au mineralization, as they are rich in chalcophile elements, have LOI < 1.8, Sr/Y > 41.8, and SiO2 63.9–65.78 wt%. This fertile magma has moderate to strong oxidation and high water contents. Accordingly, it effectively extracts chalcophile elements from the source area.
- Occurrences of a skarn-type base and precious-metal mineralization are close to the study area and may be shared by the same granitic intrusions that led the authors to suggest that the studied mineralizations are porphyry Cu- with minor Au -linked to skarn-type mineralization in larger magmatic-hydrothermal systems, like Late Paleozoic porphyries/skarns in the Central Asian Orogenic Belt.

## Data Availability

The datasets used and/or analysed during the current study are available from the corresponding author on reasonable request.
